# Supplementary material for: Emergent mechanics of a networked multivalent protein condensate
Source: Nat Commun. 2025 Jun 5;16:5237. doi: 10.1038/s41467-025-60345-9 (PMC12141447; doi:10.1038/s41467-025-60345-9)
Supplement: Supplementary file 1 — Supplementary Information [file 41467_2025_60345_MOESM1_ESM.pdf]

Supplementary Information for: Emergent mechanics of a networked multivalent protein condensate

Zhitao Liao,[1,#] Bowen Jia,[2,#] Dongshi Guan,[3,4,*] Xudong Chen,[2] Mingjie Zhang,[2,5,*] Penger Tong[1,*]
[1] *Department of Physics, Hong Kong University of Science and Technology, Clear Water Bay, Kowloon, Hong Kong*
[2] *Division of Life Science, Hong Kong University of Science and Technology, Clear Water Bay, Kowloon, Hong Kong*
[3] *State Key Laboratory of Nonlinear Mechanics, Institute of Mechanics, Chinese Academy of Sciences, Beijing 100190, China*
[4] *School of Engineering Science, University of Chinese Academy of Sciences, Beijing 100049, China*
[5] *School of Life Sciences, Southern University of Science and Technology, Shenzhen, 518055, China*
[#] *Equal contributions; * Corresponding emails: dsguan@imech.ac.cn, zhangmj@sustech.edu.cn, penger@ust.hk.*
(Dated: May 2, 2025)

I. SUPPLEMENTARY METHODS

A. Fluorescent imaging and fluorescence recovery after photo-bleaching (FRAP)

In the FRAP assay, the fluorescence recovery is assumed to be caused by diffusion of mobile proteins. Because of the laser bleaching column, the diffusion in the confocal plane has cylindrical symmetry with a radius r, so that a widely-used 2D recovery model is adopted to fit the FRAP data [1, 2],

$$I(t) = A \exp\left(-\frac{\tau}{2t}\right)\left[I_0\left(\frac{\tau}{2t}\right) + I_1\left(\frac{\tau}{2t}\right)\right], \tag{S1}$$

where I_0 and I_1 are zeroth and first order modified Bessel function, $\tau = r^2/D$ is the characteristic time of recovery, and A is the saturation value. By fitting the measured FRAP to Eq. (S1), we obtain the values of the two fitting parameters; one is the diffusion coefficient D and the other is the saturation value A for the labeled protein.

Figure S1 shows that all the normalized FRAP curves for six different protein components collapse onto a single master curve, once the normalized variables, $I(t)/A$ and τ/t, are used in the plot, indicating that the 2D diffusion model shown in Eq. (S1) agrees well with the FRAP data. To reduce the fitting uncertainties of the saturation value A at the limit of infinite time t, we set a cut-off time $t/\tau = 3$ to calculate the mobile fraction of each protein components. As shown in Fig. S1, the growth rate of FRAP at the cut-off time $t = 3\tau$ (vertical dashed line, typically 6–60 min in real time depending on different proteins), has become negligibly small. This cut-off time also excludes the long-time variations brought by the interchange of proteins in the condensed phase between the mobile ones and immobile network.

To confirm that the mobile proteins are indeed different from their immobile counterpart, we perform a series of consecutive photo-bleaching measurements on the same droplets at a 10-min interval. When the fluorescence intensity nearly saturates after the previous bleaching, we bleach the same droplet again and observe an almost 100% recovery compared to the first bleaching. As shown in Fig. S2, the second and third FRAPs fully reproduce the first FRAP curve. In the second and third FRAP measurements, the mobile proteins exchange and recover completely while the immobile ones remain bound to the network inside the droplets. The mobile and immobile proteins rarely switch their roles in the condensate for a long period of time ($\sim$ 30 min), i.e., the diffusing proteins hardly exchange with the protein network.

To examine the effect of the bleaching area on the measured recovery time τ, we conduct simultaneous FRAP measurements on two 6xPSD droplets of different sizes with approximately the same bleaching area. One droplet is significantly larger than the bleaching area, and the other is the same as the bleaching area. Figure S3 compares the FRAP measurements conducted when the bleaching area covers a whole droplet and only a portion. The fitted recovery time τ for the whole-droplet FRAP (red curve) is about 32% smaller than when the bleaching area only covers a portion of the droplet (after a minor correction due to a slight difference in the radius r of the bleaching area). We believe that one of the experimental errors contributing to the deviation between the two FRAP measurements is that the exchange of the labeled protein molecules in/out of the droplet may cause the whole-droplet FRAP to have a slightly faster recovery time τ. Therefore, the choice of bleaching area in the FRAP measurement becomes a balancing act between increasing the signal-to-noise ratio and reducing the droplet boundary effects. While our FRAP measurements have a (20-40)% standard deviation in the measured diffusion coefficient D, as shown in Table II of the main text, the obtained mean values of D for each protein component in the condensed phase are adequate for supporting the main conclusions of the work.

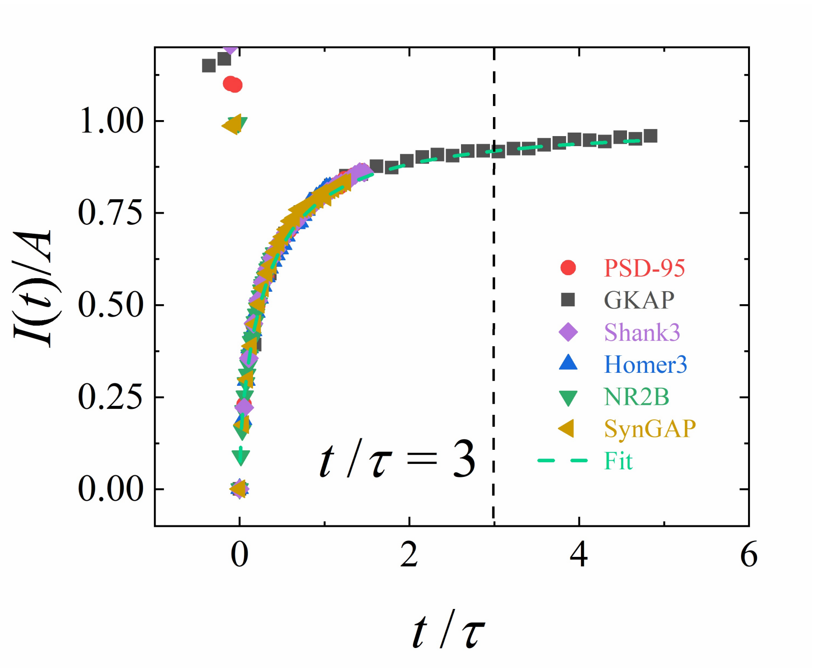

FIG. S1. **Scaling plot of the normalized FRAP data.** Normalized fluorescence intensity $I(t)/A$ as a function of the normalized time t/τ using the same data used in Fig. 3**c** of the main text. The green dashed line shows a fit of Eq. (S1) to the data points. The black vertical dashed line indicates the cut-off time $t/\tau = 3$ used to calculate the mobile fraction of proteins in 6xPSD.

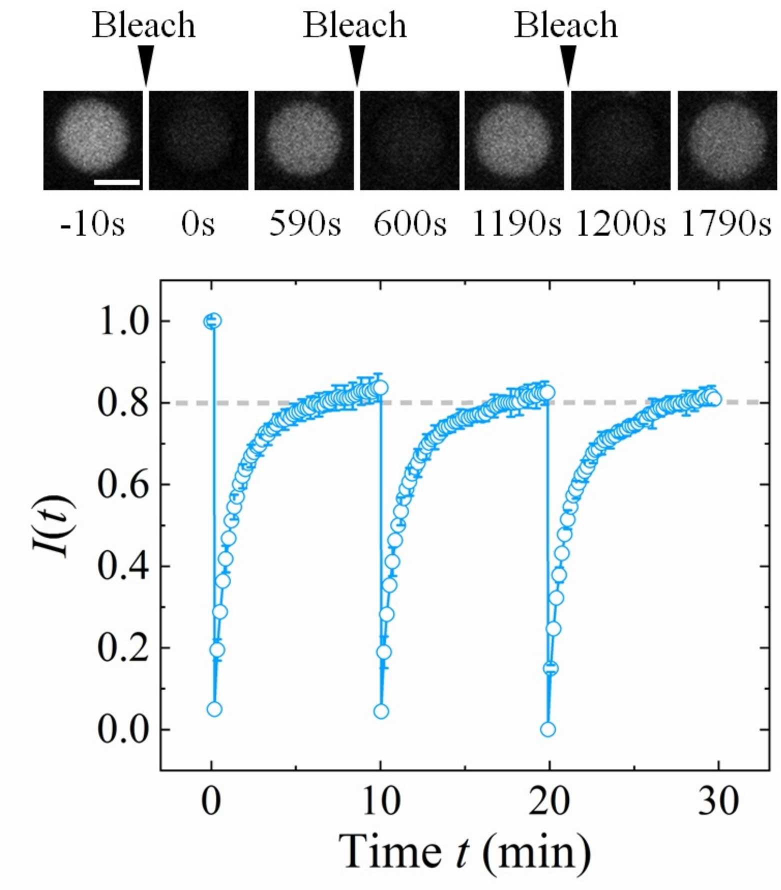

FIG. S2. **Repeated FRAP measurements on the same droplet over a 30-min period.** Time lapse images (top) and quantitative FRAP measurements (bottom) on a 6xPSD droplet with 1% PSD-95 labeled by Cy3 for three consecutive runs of photo-bleaching. The measured fluorescence intensity $I(t)$ is averaged over 7 droplets and the error bars show the standard deviation. The scale bar is 4 μm.

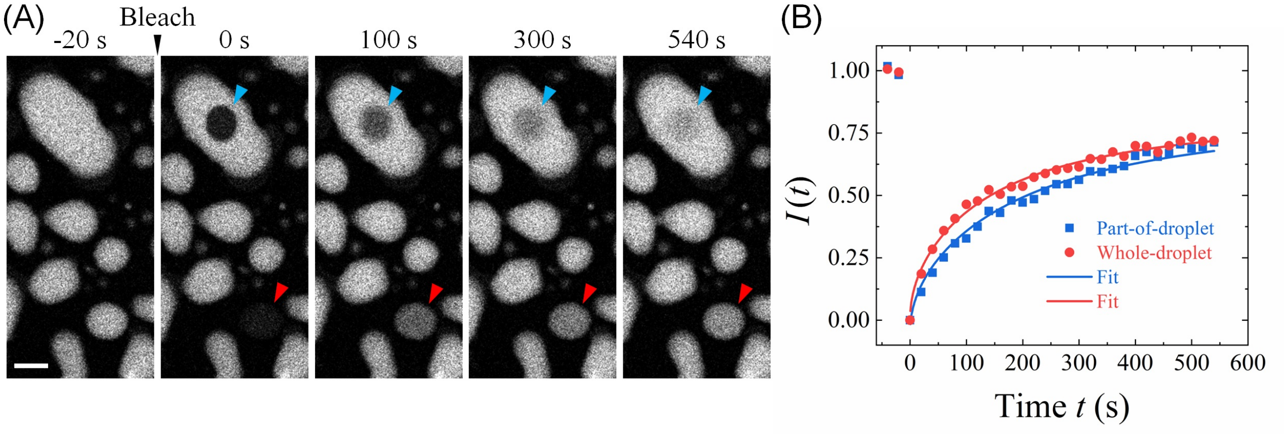

FIG. S3. **Comparison of the FRAP measurements conducted when the bleaching area covers a whole droplet and only a portion.** (A) Time-lapse images of FRAP obtained from 6xPSD droplets with 1% PSD-95 labeled by Cy3. The blue triangular arrows point to a circular bleaching area of radius 5.1 μm in a larger 6xPSD droplet. The red triangular arrows point to a circular bleaching area of radius 5.0 μm covering a whole droplet. Scale bar: 5 μm. (B) Normalized fluorescence intensity $I(t)$ that are obtained from the two bleaching areas shown in (A). The color code used is the same as that in (A). The solid lines show the fit of Eq. (S1) to the data points with $A = 0.93$ and $\tau = 619$ s for the blue curve and $A = 0.86$ and $\tau = 451$ s for the red curve.

B. Fluorescence intensity quantification (FIQ) and absolute protein concentration determination

To verify that the Cy3 label does not influence the partition of the 6xPSD condensate and the absolute fluorescence intensity measurements, we measure the fluorescence intensity of the 6xPSD droplets with different labeling ratios (see Fig. S4A). It is found that the fluorescent intensity fits well to a linear function of the labeling ratio (solid line in Fig. S4B with a zero intercept), and the obtained intensity ratios are close to the expected ones of 1:2:4. This result suggests that the Cy3 label does not affect the measured protein concentration using the FIQ assay.

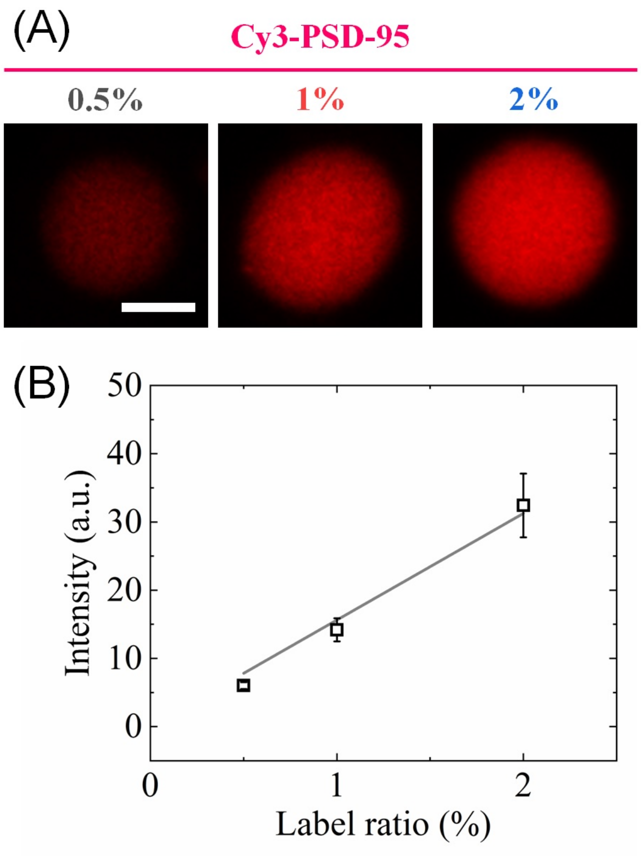

FIG. S4. **Fluorescence images and the resulting intensity of the 6xPSD droplets with different label ratios.** (A) Confocal images of the 6xPSD droplet with 0.5%, 1%, 2% PSD-95 labeled by Cy3. (B) The measured fluorescence intensity as a function of the labeling ratio. The data points are obtained, respectively, from 22 (0.5%), 28 (1%), 22 (2%) droplets. The error bars show the standard deviation of the measurements. The solid line shows a linear fit to the data points with a zero intercept.

C. Phase separation centrifuge assay

As shown in Fig. **3f** of the main text, the NR2B band obtained with Coomassie blue staining appears rather weak and vague. We then use the silver staining to improve the visualization and quantification of the NR2B band. Figure S5 shows the results for NR2B with the silver staining. In the image analysis, Fig. S5 is used to quantify the NR2B band intensity and Fig. **3f** in the main text is used to quantify the band intensity of the other five protein components.

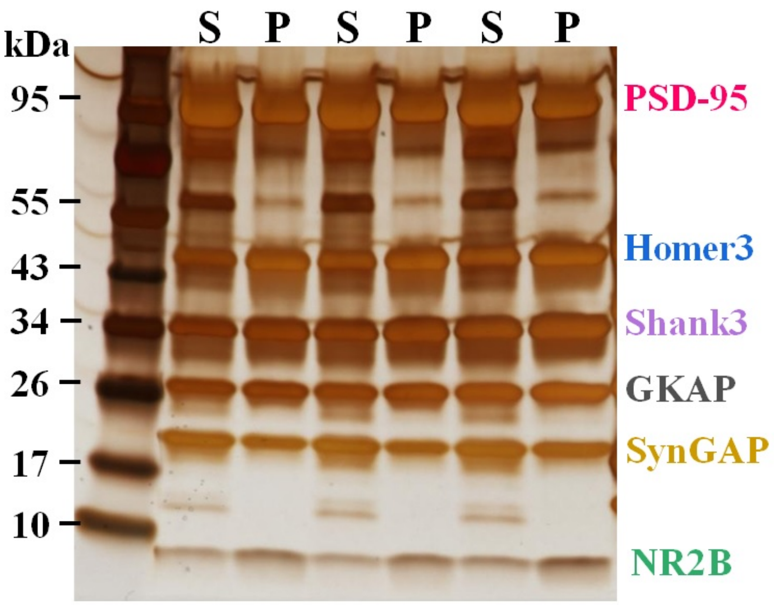

FIG. S5. **Centrifuge assay results for NR2B with Silver staining.** Three repeated runs of the centrifuge assay to separate the dilute phase (S, Supernatant) and condensed phase (P, Pellet). Protein components in both phases are then analyzed by SDS-PAGE with sliver staining.

II. SUPPLEMENTARY DISCUSSIONS

A. Atomic force microscopy (AFM) and model calculations

1. Modified Hertz model for a time-dependent modulus $E(t)$

Upon contact, the resulting force between two contact bodies is calculated by summation of stress σ over the contact region Ω. Assuming linear elasticity of the materials and small applied strain ε, one finds the stress σ acting on the elastic materials is proportional to the strain ε, namely $\sigma = E\varepsilon$, with E being the elastic modulus. In this case, the total force is given by

$$F = \int_{\Omega} \sigma \mathrm{d}A = E \int_{\Omega} \varepsilon \mathrm{d}A = E f(\delta), \tag{S2}$$

where $\mathrm{d}A$ is the differential area and $f(\delta)$ is a function of indentation depth δ, and its functional form depends on the contact geometry. When a material is viscoelastic, its modulus $E(t)$ becomes time-dependent and so does the resulting force $F(t)$, which depends on the contact history during the mechanical measurement. Using Ting's theory of viscoelastic contact, the force-indentation relation is modified because of the time-dependent $E(t)$ [3–5],

$$F(t) = \int_0^t E(t-t') \frac{\partial f(\delta(t'))}{\partial t'} \mathrm{d}t'. \tag{S3}$$

Equation (S3) calculates the cumulative viscoelastic response by an integration over the entire contact history from 0 to t.

For two compressing spheres of radius R_1 and R_2 with a Hertzian contact, one has [6]

$$f(\delta) = \frac{4R^{1/2}}{3(1-\nu^2)} \delta^{3/2}, \tag{S4}$$

where $R = 1/(1/R_1 + 1/R_2)$ is the effective radius at the contact and ν is the Poisson ratio. As most biological materials are treated as incompressible because of their high content of water, their Poisson ratio $\nu = 0.5$. Equation (S3) then becomes

$$F(t) = \frac{4R^{1/2}}{3(1-\nu^2)} \int_0^t E(t-t') \frac{\partial \delta^{3/2}}{\partial t'} \mathrm{d}t'. \tag{S5}$$

Equation (S5) describes the force-indentation relation for a viscoelastic contact as a function of time t.

With Eq. (S5), we first calculate the force relaxation curve $F(t)$ with the loading protocol as shown in Fig. S6A. This is the "approach-and-hold" protocol, as illustrated in Fig. 1**d** of the main text. Since the indentation $\delta(t)$ becomes a constant after $t > t_m$, the integration over the time $t > t_m$ does not contribute to $F(t)$. In this case, Eq. (S5) can be evaluated analytically [3],

$$\begin{aligned} F(t) &= \frac{8}{3} R^{1/2} v^{3/2} \int_0^{t_m} E(t-t') \sqrt{t'} \mathrm{d}t' + 0 \\ &\simeq \frac{16}{9} R^{1/2} (v t_m)^{3/2} E_0 \\ &\cdot \left\{ C_1 \frac{3\tau_1}{4t_m} \left[2e^{\frac{t_m}{\tau_1}} - \sqrt{\frac{\pi\tau_1}{t_m}} \mathrm{Erfi}\left(\sqrt{\frac{t_m}{\tau_1}}\right) \right] e^{-t/\tau_1} + C_2 \cdot {}_2F_1\left[\frac{3}{2}, \alpha; \frac{5}{2}; \frac{t_m}{t+\tau_2}\right] \left(1 + \frac{t}{\tau_2}\right)^{-\alpha} \right\}, \end{aligned} \tag{S6}$$

where $\mathrm{Erfi}(x)$ is the imaginary error function and ${}_2F_1[x]$ is the hypergeometric function. At the fast loading limit with $t_m \to 0$, Eq. (S6) is simplified to

$$\begin{aligned} F(t) &\simeq \frac{16}{9} R^{1/2} E_0 \delta^{3/2} \left[C_1 e^{-t/\tau_1} + C_2 (1 + t/\tau_2)^{-\alpha} \right] \\ &= F_0 \left[C_1 e^{-t/\tau_1} + C_2 (1 + t/\tau_2)^{-\alpha} \right]. \end{aligned} \tag{S7}$$

This result explains why the normalized force relaxation curve $F(t)/F_0$, as shown in Fig. 1**e** of the main text, remains the same as the relaxation modulus $E(t)$ when the highest loading speed $v \simeq 100$ μm/s is used.

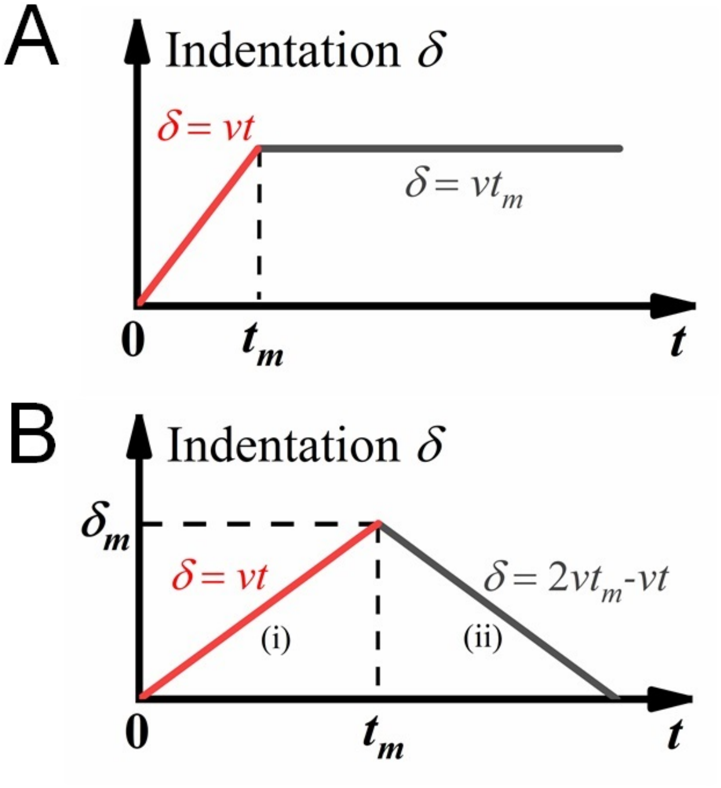

FIG. S6. **Controlled indentation protocols during the mechanical measurements.** A sketch showing how the indentation $\delta(t)$ varies with time t during (A) the stress relaxation measurement and (B) force-indentation measurement. The relaxation measurement is made with the colloidal probe moving at a constant speed $v \simeq 100$ μm/s and then the probe stops at $t = t_m$ and its position is kept at a constant indentation $\delta = vt_m$. In the force indentation measurement, the colloidal probe moves forward and backwards from the droplet surface at a constant speed v.

If a lower loading speed is used in the force relaxation measurement, Eq. (S6) instead of Eq. (S7) should be used to describe the force relaxation curve, which has a set of five relaxation parameters: C_1, C_2, τ_1, τ_2, and α, together with the loading time t_m, which can be measured in the experiment. Figure S7 shows the measured force relaxation curves $F(t)/F_0$ at three different loading speeds (colored symbols) and the calculated $F(t)/F_0$ (grey lines) using Eq. (S6). The five relaxation parameters, C_1, C_2, τ_1, τ_2, and α, are determined from the high-speed data ($v = 100$ μm/s) using Eq. (S7). It is seen that the speed-dependence of the measured $F(t)/F_0$ from a single 6xPSD droplet is well described by Eq. (S6) with a unique power-law exponent $\alpha \simeq 0.5$. The slight deviations at short times t between the measured $F(t)/F_0$ at $v = 10$ μm/s and the calculated $F(t)/F_0$ might be caused by the experimental uncertainties in determining the loading time t_m.

Next, we use Eq. (S5) to calculate the force-indentation curve $F(\delta)$ with the loading protocol as shown in Fig. S6B. This is the continuing-approach-at-constant-speed" protocol, as illustrated in Fig. 1**g** of the main text. With $\delta(t) = vt$, Eq. (S5) becomes [3]

$$
\begin{aligned}
F(\delta, t) \simeq & \frac{16}{9} R^{1/2} E_0 \delta^{3/2} \cdot \left\{ \frac{3}{2} C_1 \left[\frac{\tau_1}{t} - \frac{\sqrt{\pi}}{2} \left(\frac{\tau_1}{t} \right)^{\frac{3}{2}} e^{-\frac{t}{\tau_1}} \mathrm{Erfi} \left(\sqrt{\frac{t}{\tau_1}} \right) \right] + C_2 \frac{3\sqrt{\pi}\Gamma(1-\alpha)}{4\Gamma(\frac{5}{2}-\alpha)} \left(\frac{t}{\tau_2} \right)^{-\alpha} \right\} \\
= & \frac{16}{9} R^{1/2} E_0 \delta^{3/2} C(t),
\end{aligned}
\tag{S8}
$$

where we have assumed $t/\tau_2 \gg 1$ to simplify the calculation of the power law contributions. The time-dependent factor $C(t)$ in the brackets includes all the contributions from the relaxation modulus. Using the five relaxation parameters, C_1, C_2, τ_1, τ_2, and α, obtained from the high-speed force relaxation measurements (at $v = 100$ μm/s), the factor $C(t) = C(\delta/v)$ is uniquely determined as a function of δ, where v is the indentation speed used in the force-indentation measurements. By dividing out the function $C(\delta/v)$ from the measured force-indentation curves $F(\delta)$ at different loading speeds, we obtained the speed-independent force-indentation curves $F(\delta)/C(\delta/v)$, which collapse onto a single master curve as shown in Fig. 1**i** of the main text.

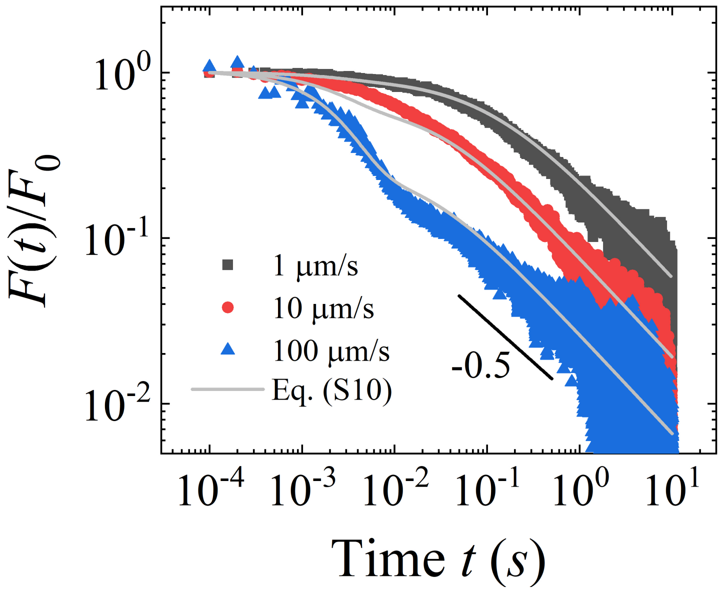

FIG. S7. **Force relaxation curves obtained from a single 6xPSD droplet at different loading speeds v.** The normalized force $F(t)/F_0$ as a function of time t is obtained at three loading speeds: $v = 1$ μm/s (black squares), 10 μm/s (red circles), and 100 μm/s (blue triangles). The data are obtained from a single 6xPSD droplet. The gray solid lines are the calculated $F(t)/F_0$ using Eq. (S6) with the same set of five relaxation parameters (C_1, C_2, τ_1, τ_2, and α) but different values of t_m. The black solid line indicates the power-law decay, $t^{-0.5}$.

2. Finite thickness correction

For most force-indentation measurements, one usually assumes the materials under compression are semi-infinite, since the indentation depth δ is often much smaller than the sample thickness h, i.e., $\delta/h \ll 1$. However, the 6xPSD droplets are thin and their value of δ/h is often not negligible. In this case, the effect of finite thickness may lead to an overestimation of the initial modulus E_0, because the probe can also sense the rigid glass substrate that exerts extra elastic forces. To correct the finite thickness effect, we consider a widely-used model that describes the contact mechanics between a stiff sphere (probe) and an elastic sample of finite thickness h under the non-bonded boundary condition (6xPSD droplets are not bonded to the glass substrate) [7, 8]

$$F \simeq \frac{16}{9} E_0 R^{1/2} \delta^{3/2} \cdot \begin{cases} 1 + 0.88\chi + 0.78\chi^2 + \ldots, & \chi < 0.8 \\ 1.18\chi, & \chi > 0.8 \end{cases} \tag{S9}$$

where $\chi = \sqrt{R\delta}/h$ is a dimensionless factor defined as a ratio of impact region to the droplet thickness. Equation (S9) quantifies the contribution of the stiff substrate to the probed force, which increases as the sample becomes thinner, as expected. In the limit of $\chi \to 0$, the resulting elastic force recovers back to the Hertzian model. To ensure the convergence of the series in Eq. (S9), we omit the higher order terms (χ^3 and beyond), which does not alter the final results significantly.

For viscoelastic materials, such as living cells, we assume the factor $C(t)$ in Eq. (S8) does not change much with the sample thickness, as the effect of substrate is static. In this case, Eq. (S9) becomes

$$\frac{F(\delta, t)}{C(t)} \simeq \frac{16}{9} E_0 R^{1/2} \delta^{3/2} \cdot \begin{cases} 1 + 0.88\chi + 0.78\chi^2 + ..., & \chi < 0.8 \\ 1.18\chi, & \chi > 0.8 \end{cases} \tag{S10}$$

In the experiment, Eq. (S10) is used to calculate the absolute value of modulus E_0, as shown in Table I of main text. The dimensionless factor χ is determined by using the actual indentation depth δ and the measured thickness h of the individual droplets by AFM.

3. Estimation of mesh size ξ_{net}

With the measured immobile protein concentration n_{im}, one can find the average volume occupied by each network molecule $v_{\mathrm{net}} = 1/(N_A n_{\mathrm{im}})$, where N_A is the Avogadro's constant. If this volume is assumed to be a sphere with radius R_m, we have $R_m = \sqrt[3]{3v_{\mathrm{net}}/4\pi} = \sqrt[3]{3/(4\pi N_A n_{\mathrm{im}})}$. The mesh size (or the correlation length) ξ_{net} can be estimated as the average distance between two adjacent network molecules, namely $\xi_{\mathrm{net}} \simeq 2R_m = 2\sqrt[3]{3/(4\pi N_A n_{\mathrm{im}})}$. This simple way of estimating the network mesh size has been used in previous studies [9–11].

B. Complex shear modulus $G^*(\omega)$ of the protein condensates

1. Relation between the shear modulus $G^*(\omega)$ and relaxation modulus $E(t)$

As mentioned in the main text, the Fourier transform of the relaxation modulus $E(t)$ is directly linked to the complex shear modulus $G^*(\omega)$. Specifically, we have [12, 13]:

$$G^*(\omega) = \frac{i\omega}{2(1+\nu)} \int_0^\infty E(t) e^{-i\omega t} \mathrm{d}t \tag{S11}$$

where ν is the Poisson ratio. Because of their high water content, the protein condensates are usually treated as incompressible in volume and thus $\nu = 0.5$. Equation (S11) states that $E(t)$ and $G^*(\omega)$ are two equivalent and complementary quantities of characterizing the viscoelastic materials; one is in time domain and the other is in frequency domain. In Fig. 1**f** of the main text, we present the data in the time domain, because they are directly measured by AFM. Since the two relaxation modes in Eq. (2) of the main text are well separated in time, using $E(t)$ is more convenient for identifying the molecular origins of the condensate's viscoelasticity.

We now convert the measured $E(t)$ to the complex modulus $G^*(\omega)$ using Eq. (S11) and discuss the viscoelastic property of the protein condensates in the frequency domain. We will compare our results with those obtained from previous studies [14, 15]. The complex modulus, $G^*(\omega) = G'(\omega) + iG''(\omega)$, contains the real-part storage modulus

$G'(\omega)$ and the imaginary-part loss modulus $G''(\omega)$, which are used to characterize, respectively, the elastic and viscous properties of the material. Experimentally, $G^*(\omega)$ is often obtained using an oscillatory shear rheometer or by particle tracking methods (micro-rheology). Here, we adopt a widely used algorithm [16–18] to convert $E(t)$ to $G^*(\omega)$.

For a Maxwell fluid, its storage modulus $G'(\omega)$ and loss modulus $G''(\omega)$ take the form [13, 19]

$$G'(\omega) = G_0 \frac{\omega^2\tau_1^2}{\omega^2\tau_1^2+1}, \quad G''(\omega) = G_0 \frac{\omega\tau_1}{\omega^2\tau_1^2+1}, \tag{S12}$$

where G_0 is the modulus amplitude and τ_1 is the crossover time. At the low frequency limit, the two moduli scale as $G'(\omega) \sim \omega^2$ and $G''(\omega) \sim \omega$, respectively. It is seen from Fig. 2**a** of the main text that the obtained $G'(\omega)$ and $G''(\omega)$ for PGL-3 are well described by Eq. (S12) and scale as $G'(\omega) \sim \omega^2$ and $G''(\omega) \sim \omega$ at low frequencies. The data show small deviations from the predicted scaling, $G''(\omega) \sim \omega$, at very low frequencies, because the measured force relaxation $E(t)$ at long times t has reached the noise level, i.e., the value of the resulting $G''(\omega)$ is too small to be measured accurately. Despite the slight deviations, the fittings shown in Fig. 2**a** of the main text give the plateau modulus $G_0 \simeq 455$ Pa and the crossover time $\tau_1 \simeq 2.1$ ms. The corresponding value of the effective condensate viscosity is $\eta \simeq G_0(\tau_1/2\pi) \simeq 0.15$ Pa·s. These fitting results are in good agreement with the reference values of $G_0 \simeq 500$ Pa, $\tau_1 \simeq 3$ ms, and $\eta \simeq 0.24$ Pa·s, as reported in Ref. [14] for fresh PGL-3 samples. Figure 2**a** in the main text thus verifies that the PGL-3 condensate is of Maxwell type and our AFM methodology of measuring the relaxation modulus $E(t)$ works.

At the long time limit, we find from Eq. (2) in the main text that $E(t) \simeq E_2(t/\tau_2)^{-\alpha}$. The corresponding $G^*(\omega)$ in Eq. (S11) at the low frequency limit then takes the form

$$G^*(\omega) \simeq G_0\Gamma(1-\alpha)(\omega\tau_2)^\alpha \left[\cos\left(\frac{\pi}{2}\alpha\right) + i\sin\left(\frac{\pi}{2}\alpha\right)\right], \tag{S13}$$

where $G_0 = E_2/[2(1+\nu)]$ and $\Gamma(x)$ is the Gamma function. Equation (S13) states that at the low frequency limit, the storage modulus $G'(\omega)$ and loss modulus $G''(\omega)$ follow the same power-law scaling: $G'(\omega) \sim (\omega\tau_2)^\alpha$ and $G''(\omega) \sim (\omega\tau_2)^\alpha$, but their amplitude ratio varies with α as $G''(\omega)/G'(\omega) = \tan(\frac{\pi}{2}\alpha)$. In this case, $\alpha = 1/2$ becomes a crossover value, below which the network behaves more like an elastic solid ($G''(\omega)/G'(\omega) < 1$) and above which the network behaves more like a viscous fluid ($G''(\omega)/G'(\omega) > 1$).

While the measured $G'(\omega)$ and $G''(\omega)$ for the 6xPSD condensate cannot be fitted to the Maxwell model, one can still find the plateau modulus $G_0 \simeq 3517$ Pa from the measured $G'(\omega)$ and the crossover time $\tau_1 \simeq 3.8$ ms between the measured $G'(\omega)$ and $G''(\omega)$. Hence, the effective viscosity of the 6xPSD condensate is estimated as $\eta \simeq G_0(\tau_1/2\pi) \simeq 2.1$ Pa·s. This value of η for the 6xPSD condensate is about 14 times larger than that of the fresh PGL-3 condensate.

2. *Relaxations in protein condensates*

As mentioned in the introduction of the main text, the simple one- or two-component protein condensates are well described by the Maxwell model for homogeneous polymeric fluids without a percolating network (also called Maxwell fluids). As shown in Fig. 1**f** of the main text, the relaxation modulus $E(t)$ of the model protein condensate, PGL-3, follows the simple exponential relaxation, $E(t) \simeq E_1 \exp(-t/\tau_1)$, with the relaxation time $\tau_1 = 2.8$ ms. With the microscopic picture given in the Discussion section of the main text, the diffusive relaxation time for a simple protein condensate is $\tau_1 \simeq \xi_{\rm mo}^2/D$, where $\xi_{\rm mo}$ is the size of the cage formed by the surrounding molecules in the condensate and D is their diffusion coefficient. Using the same fluorescence intensity quantification (FIQ) method as described in the main text, we quantify the fluorescence intensity of the PGL-3 droplets and obtain the PGL-3 concentration $n_{PGL3} = 1.02 \pm 0.13$ mM. The corresponding value of ξ_{mo} is $\xi_{mo} \simeq 14.6$ nm. With the estimated value of $D_{PGL3} \simeq 0.05$ μm^2/s for PGL-3, which was obtained from the FRAP experiments in Refs. [14, 20, 21], we find the diffusive relaxation time for PGL-3 to be $\tau_1 \simeq \xi_{mo}^2/D_{PGL3} = 4.3$ ms. The estimated value of τ_1 is close to the measured value of $\tau_1 = 2.8$ ms.

Since each protein molecule has a thermal energy k_BT, the modulus amplitude E_1 should scales as $E_1 \simeq k_BT/\xi_{\rm mo}^3$ [13, 19], and the diffusion coefficient goes as $D \simeq k_BT/(6\pi\eta\xi_{\rm mo})$. Therefore, we have $\tau_1 \simeq \xi_{\rm mo}^2/D \simeq 6\pi\eta/E_1 = 2\pi\eta/G_0$, where $G_0 = 2(1+\nu)E_1$ is the shear modulus with $\nu = 1/2$ being the Poisson ratio for incompressible fluids. The above relation can be written in a more familiar form $\eta \simeq G_0[\tau_1/(2\pi)]$. Theses results further indicate that our argument for the diffusive relaxation time τ_1 is fully consistent with the phenomenological model for Maxwell fluids.

When a percolating network is formed in the protein condensate, the deformed network under compression has two possible relaxation channels to release the accumulated strain (or stress). One is the power-law relaxation, $E_2(1+t/\tau_2)^{-\alpha}$, used to characterize the slow reorganization of soft glassy networks, which have a broad spectrum of

relaxation times because of structural disorder and metastability [22]. In particular, the model of cross-link-governed dynamics (CGD) [23] for transient protein networks predicts that $\alpha = 1/2$ and that the power-law relaxation starts at time $t \gtrsim \tau_2$, where τ_2 is the characteristic time for a cross-linker to bind and unbind (see the main text for more discussions).

The other relaxation is called poroelastic relaxation, resulting from the solvent movement out of the porous matrix in the compressed region of the network [24]. In the poroelastic model, the imposed mechanical compression by the AFM probe generates a pressure gradient in the porous matrix [25], which forces the fluid (or solvent) to move outward diffusively across the impact region of radius a (see Fig. S9(B) below for the probe-droplet contact geometry). For a Hertzian contact, one has $a = \sqrt{R\delta}$. As a result, the normalized force relaxation $F(t)/F_0$ decays exponentially, $F(t)/F_0 \simeq \exp(t/\tau_p)$, at short times. The relaxation time τ_p is given by $\tau_p \simeq a^2/D_p$ [24], where the effective diffusion coefficient D_p in the porous matrix goes as $D_p \sim \xi_{\rm net}^2 E_2/\eta$. Here $\xi_{\rm net}$ is the mesh size of the network and E_2 is the Young's modulus of the network [24]. Therefore, we have $\tau_p \simeq (R\delta/\xi_{\rm net}^2)(\eta/E_2)$.

Because the mesh size $\xi_{\rm net}$ is usually much smaller than the impact region size a, the poroelastic relaxation time τ_p is thus much larger than the diffusive relaxation time τ_1 of the protein condensates, which is of the order of η/E_1, as discussed above. For the 6xPSD condensate and the setup used in the experiment, we have the probe radius $R \simeq 7.5$ μm, indentation $\delta \simeq 0.3$ μm (see Fig. 1**h** in the main text), network mesh size $\xi_{\rm net} \simeq 12.3$ nm, and thus the length ratio, $R\delta/\xi_{\rm net}^2 \simeq 15 \times 10^3$. Since E_2 is approximately equal to E_1 for the 6xPSD condensate (see Table I in the main text), we have $\eta/E_2 \simeq \tau_1/(6\pi)$, as discussed above. In this case, the poroelastic relaxation time becomes $\tau_p \simeq 10^3\tau_1$. With the measured $\tau_1 \simeq 2.8$ ms (see Table I in the main text), we have $\tau_p \simeq 2.8$ s. Similar values of τ_p were also reported in recent studies [3, 24, 26].

The actual relaxation of the protein network is determined by the relaxation channels with the fastest relaxation times. An important assumption that the poroelastic model made is that the porous matrix of the medium remains stable and does not relax during the poroelastic transport [25]. In other words, the lifetime of the protein network itself needs to be longer than the poroelastic relaxation time τ_p. This may be true for certain polymeric networks, such as strong hydrogels and rubber-like materials, in which the lifetime of their cross-links is longer than τ_p. The protein network in the 6xPSD condensate, on the other hand, is weak and dynamic and its cross-links bind and unbind continuously over a range of times, which gives rise to a separate stress relaxation channel that allows the network to flow at long times [23, 27–29].

As shown in Fig. 1**f** of the main text, the measured $F(t)/F_0$ (i.e., the accumulated stress) has decayed more than 95% through the power-law relaxation over a time span of 0.01–1 s, which is less than τ_p. This result suggests that, before reaching τ_p, the applied stress has actually relaxed primarily through local concentration relaxation and network rearrangements via dynamic binding and unbinding in the condensate. Therefore, the poroelastic model does not apply to transient protein networks, such as that in the 6xPSD condensate, whose lifetime is shorter than the poroelastic relaxation time τ_p.

As discussed above, the main difference between poroelastic and local concentration relaxation lies primarily in the effective distance, through which the solvent or solute (protein) relaxes by diffusion. In the poroelastic model, the solvent relaxation under the mechanical compression takes place over the impact region of radius $a \simeq \sqrt{R\delta}$, which increases with indentation δ. To further test this effect, we perform additional force relaxation measurements with varying applied force amplitude F_0. The increase in F_0 gives rise to an increase in δ and hence an increase in $a^2 \simeq R\delta$. Figure S8 shows how the measured exponential relaxation time τ_1 changes with δ. It is seen that the obtained values of τ_1 from different 6xPSD droplets (different colors) do not show any systematic variations when the imposed indentation δ is changed nearly five times. The measured values of τ_1 scatter in the range of 4 ± 1 ms (the error bar gives a measure of droplet-to-droplet variations) and do not show a clear linear dependence on δ, as expected for poroelastic relaxation. Figure S8 thus confirms that the exponential relaxation in the measured $F(t)/F_0$ for the 6xPSD droplets is not associated with poroelastic relaxation.

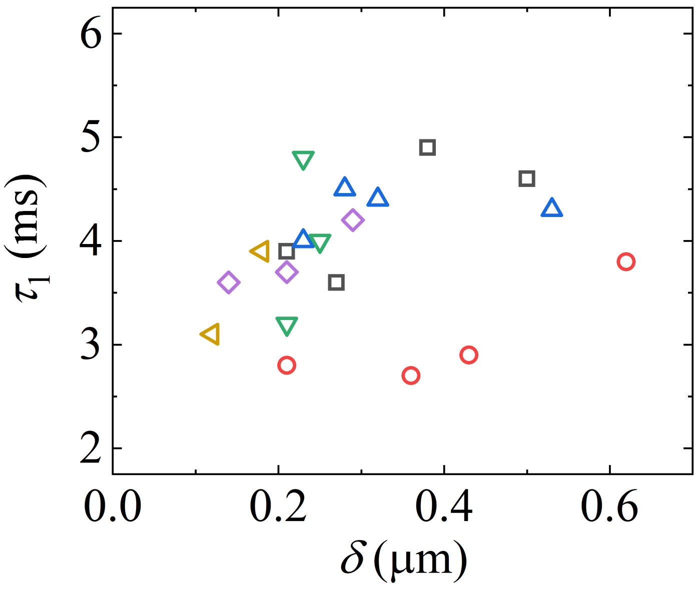

FIG. S8. **Obtained exponential relaxation time τ_1 as a function of the imposed indentation δ.** The relaxation time τ_1 is obtained from the measure force relaxation curves $F(t)/F_0$ for different values of the applied force amplitude F_0. Color-coded data points are obtained from different 6xPSD droplets ($N = 6$).

C. Contact geometry between the AFM probe and upper surface of the condensate droplet

With the AFM setup as shown in Fig. 1**b** of the main text, we are unable to directly observe the contact area and its evolution during the force relaxation and force-indentation measurements. To examine the contact geometry between the AFM probe and upper surface of the condensate droplet, we design a new contact visualization apparatus using confocal microscopy together with a micro-manipulation probe. Figure S9(A) shows the experimental setup. The micro-manipulation probe contains a micron-sized glass fiber (probe holder) with its tip forged into a glass sphere. Alternatively, one may glue a glass bead to the fiber tip. A three-axis micro-manipulator driven by stepper motors is used to control the motion of the probe. The 6xPSD condensate solution is placed on a coverslip for imaging and the probe can move around in the solution until it touches a desired condensate droplet. To visualize the probe-droplet contact geometry, we use confocal microscopy to capture the time-lapse and z-stack images of the droplet in contact with the probe having a z-interval of 0.5 μm. The 6xPSD condensate droplets are labelled with a fluorescent dye Cy3, as described in Methods section of the main text.

Figure S10 shows, respectively, the bright field images (left column), confocal fluorescent images (middle column), and 3D confocal reconstruction images (right column) of a 6xPSD condensate droplet in contact with the probe. The three rows of images ((A), (B), and (C)) are taken, respectively, before the probe touches the droplet (before contact), right after the contact, and 7 s after the contact. A key feature for the probe-droplet contact is that the contact edge appears as a bright circular ring in the bright field image (pointed by the white arrows in (B) and (C)), whereas the contact region appears as a dark dent in the confocal image (pointed by the white arrows in (B) and (C)). The contact region (i.e., the dark dent) is clearly seen in the 3D confocal reconstruction images.

Figure S11 shows, respectively, the evolution of the confocal fluorescent images and fluorescence intensity profiles across the yellow line at different z positions. The three sets of data ((A), (B), and (C)) are obtained, respectively, before the contact, right after the contact, and 7 s after the contact. The contact region gives rise to an intensity drop in the middle of the fluorescence intensity profile (see the blue arrows and red bars in the Fig. S11). By comparing the data obtained 7 s after the contact (Fig. S10(C) and Fig. S11(C)) with those obtained right after the contact (Fig. S10(B) and Fig. S11(B)), we find that there is no any significant change in the contact area (see more discussions below).

From the obtained confocal images in Figs. S10 and S11, we now extract quantitative parameters associated with the contact geometry, as shown in Fig. S9(B). First, with the probe radius $R_p = 10$ μm and droplet radius $R_d = 13$ μm, we find the reduced radius $R = 1/(1/R_p + 1/R_d) \simeq 5.7$ μm. Second, the indentation δ contains two parts: $\delta = \delta_1 + \delta_2$, with the bottom of the rectangular imaging section (marked by the dashed lines in Fig. S9(B)) as the dividing line. Here, δ_1 is the distance from the top of the droplet to the imaging section bottom, and δ_2 measures the distance from the imaging section bottom to the bottom of the probe.

To determine δ_1, one needs to know the z-position $z_{\rm top}$ of the upper surface of the droplet. Here we define the z-position $z_{\rm top}$ as that at which the fluorescence intensity drops to one half of the intensity at the droplet center. We then obtain the value of $z_{\rm top}$ by examining how the fluorescence intensity changes with z. The z-position $z_{\rm btm}$ of the imaging section bottom marks the beginning position of the 3D confocal reconstruction image, which can be found directly from the imaging software. Therefore, the value of δ_1 can be calculated from the difference of the two z-positions, i.e., $\delta_1 = z_{\rm top} - z_{\rm btm} = 3.5$ μm. The value of δ_2 is determined from the geometry as, $\delta_2 \simeq R_p - \sqrt{R_p^2 - r_d^2} = 0.25$ μm, where $r_d = 2.2$ μm is the radius of the measured dark dent. Thus, we find $\delta \simeq 3.8$ μm. Third, the contact radius $a \simeq 4.8$ μm is measured directly from the bright field image for simplicity. Finally, the Hertzian contact radius is given by $a_H = \sqrt{R\delta} \simeq 4.7$ μm. which is close to the obtained value of a.

Table S1 summarizes the results of the eight geometry parameters: R_p, R_d, R, δ_1, δ_2, δ, a and a_H, obtained from three 6xPSD condensate droplets. As shown in Fig. S9(B), the contact between the probe and the condensate droplet is characterized primarily by two quantitative parameters: the total indentation δ in the vertical direction and the contact radius a in the horizontal direction. For the probe radius $R_p = 10$ μm comparable to the droplet radius R_d, we find from Table S1 that the measured contact radius a agrees with the calculated Hertzian radius a_H within the experimental uncertainties. This result suggests that the probe-droplet contact remains a Hertzian contact during the loading period. To further verify this conclusion, we show a log-log plot of the measured force-indentation curves $F(\delta)$ at different loading speeds in Fig. S12. It is seen that the measured force-indentation curves $F(\delta)$ at large indentations (away from the contact point) all follow the Hertz scaling law, $F(\delta) \propto \delta^{3/2}$, for different loading speeds (or different times after the contact).

Table S2 summarizes the results of the eight geometry parameters: R_p, R_d, R, δ_1, δ_2, δ, a and a_H, obtained at three different times: before the contact, right after the contact, and 7 seconds after the contact. It is seen from Table S2 that the contact radius a and the total indentation δ obtained 7 seconds after the contact remain essentially the same as those obtained right after the contact (within the experimental uncertainties) and no visible time dependence is observed among the contact parameters. From the results shown in Tables S1, S2, and Fig. S12, we conclude that

the probe-droplet contact during the force relaxation measurements remains unchanged and that during the loading in the force-indentation measurements exhibits a typical Hertzian contact behavior.

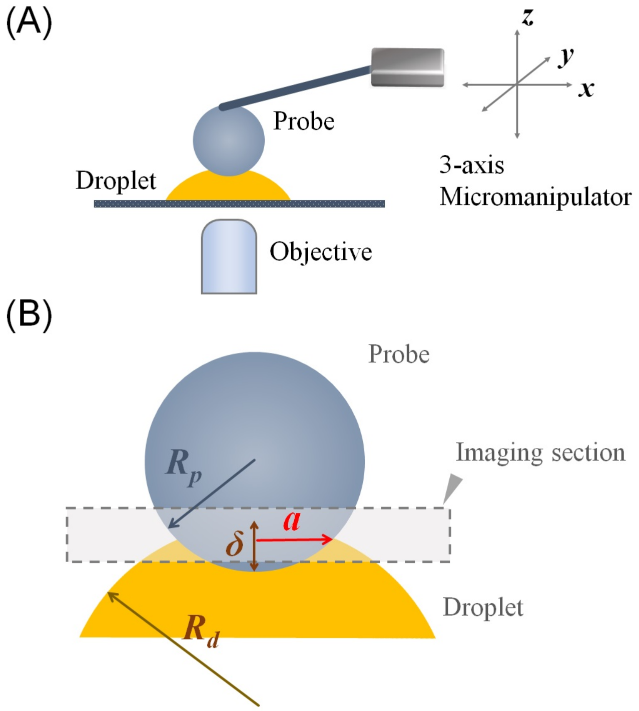

FIG. S9. **Experimental setup and probe-droplet contact geometry.** (A) Experimental setup for visualization of the probe-droplet contact and the coordinate system used in the experiment. (B) The probe-droplet contact geometry and associated parameters.

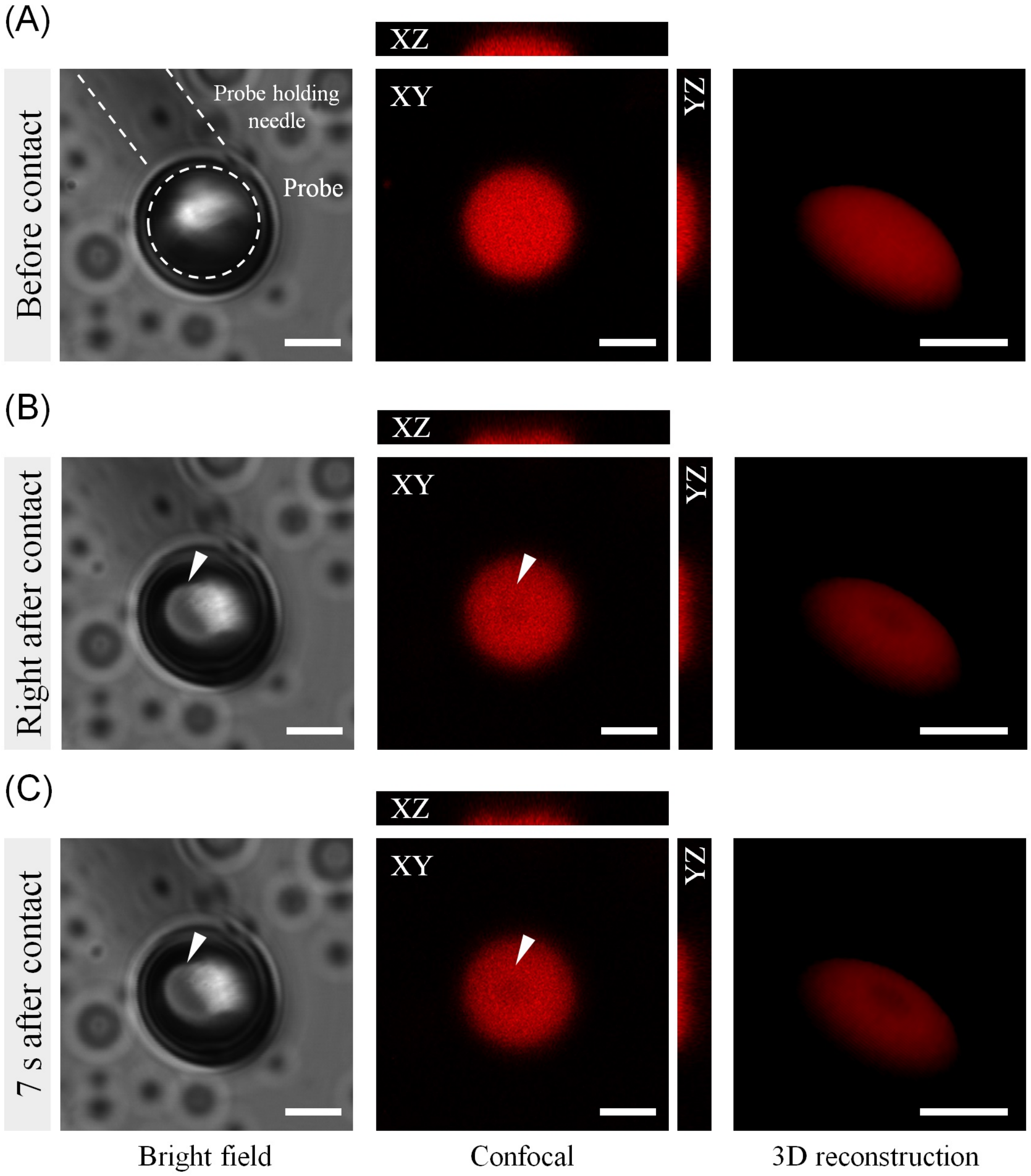

FIG. S10. **Visualization of the probe-droplet contact.** The bright field images (left column), confocal fluorescent images (middle column), and 3D confocal reconstruction images (right column) of a 6xPSD condensate droplet in contact with the probe. The 3D images are reconstructed from the stack of all the x-y images at different z positions and they are rotated to have a better view of the droplet top surface and the dent created by the indentation of the probe. The three rows of images ((A), (B), and (C)) are taken, respectively, before the probe touches the droplet (before contact), right after the contact, and 7 s after the contact. The dashed lines in (A) indicate the outer contour line of the probe and the probe holding fiber, respectively. The probe is not visible in the fluorescent image as it is not labeled. The confocal images in the middle column show the three orthogonal views of the contact region. The white arrows in (B) and (C) point to the contact region. To speed up the confocal imaging, only the top portion of the condensate droplet is imaged. All scale bars are 10 μm. Similar images were obtained in at least two separate measurements.

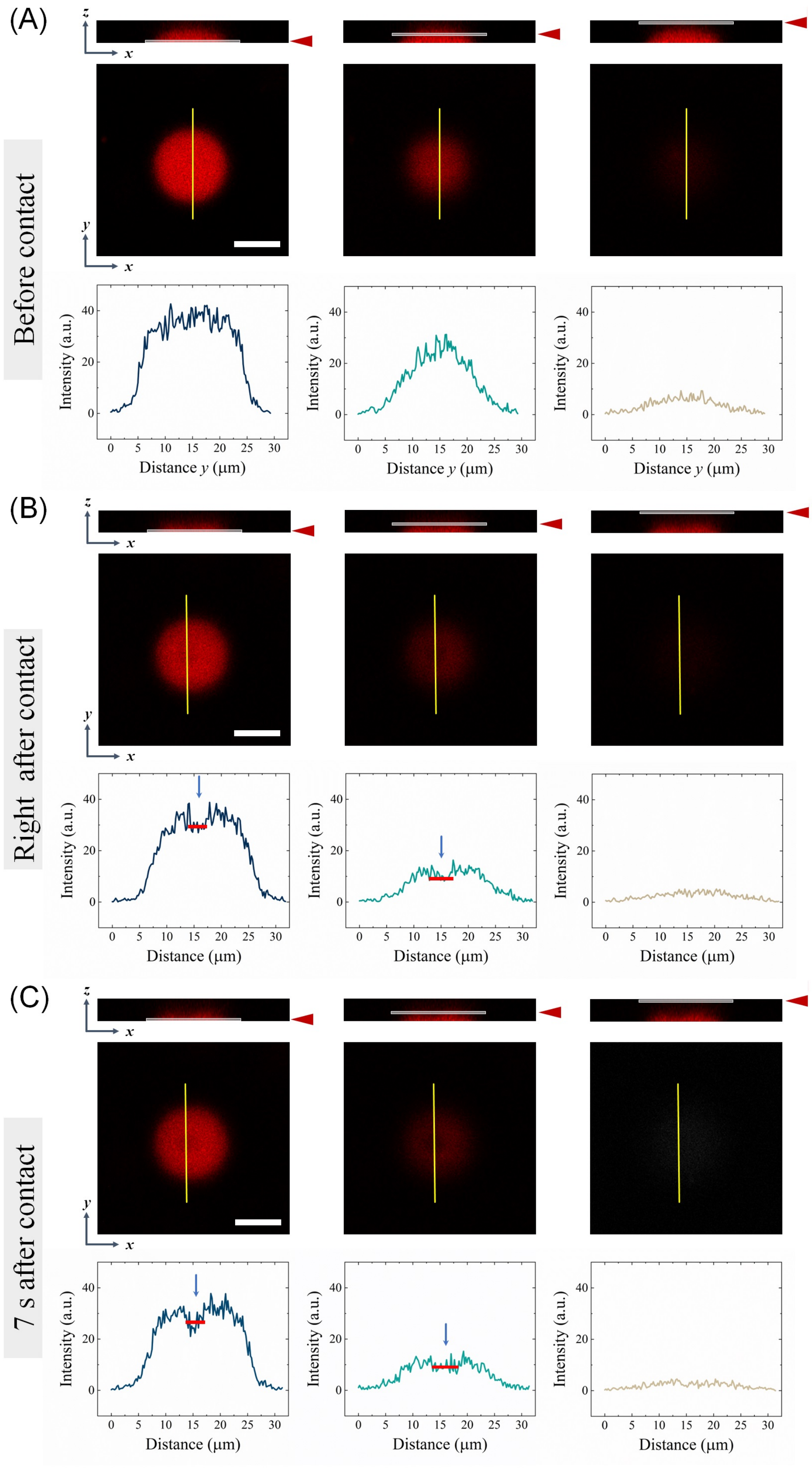

FIG. S11. **Confocal fluorescent images and fluorescence intensity profiles at different z positions.** The x-y and x-z confocal fluorescent images (upper panel) and the fluorescence intensity profiles across the yellow line (lower panel), at different z positions across the probe-droplet contact region. The red arrows and white lines in the x-z confocal images indicate the z position of the images, which increases from the left to right. The three sets of data ((A), (B), and (C)) are obtained, respectively, before the contact, right after the contact, and 7 s after the contact. The blue arrows in the lower panel of (B) and (C) point to the fluorescence drop caused by the indentation of the probe. The red bar in the intensity profiles indicates the width of the darker dent appeared in the fluorescence intensity profiles. The scale bars are 10 μm. Similar images were obtained in at least two separate measurements.

Droplet	R_p (μm)	R_d (μm)	R (μm)	δ_1 (μm)	δ_2 (μm)	δ (μm)	a (μm)	a_H (μm)
1	10.0 ± 0.5	13 ± 1	5.7 ± 0.3	3.5 ± 0.5	0.3 ± 0.5	3.8 ± 0.7	4.8 ± 1.0	4.7 ± 0.9
2	10.0 ± 0.5	14 ± 1	5.7 ± 0.3	3.0 ± 0.5	1.0 ± 0.5	4.0 ± 0.7	5.3 ± 1.0	4.8 ± 0.9
3	10.0 ± 0.5	13 ± 1	5.6 ± 0.3	3.5 ± 0.5	0.8 ± 0.5	4.3 ± 0.7	5.0 ± 1.0	4.9 ± 0.9

TABLE S1. **Measured geometry parameters at the probe-droplet contact.** As shown in Fig. S9(B), the geometry parameters at the probe-droplet contact include the probe radius R_p, the droplet radius R_d, the reduced radius $R = 1/(1/R_p + 1/R_d)$, the distance δ_1 between the upper surface of the droplet and the bottom of the imaging section, the distance δ_2 between the imaging section bottom and the bottom of the probe, the total indentation $\delta = \delta_1 + \delta_2$, the observed contact radius a, and the calculated Hertzian radius $a_H = \sqrt{R\delta}$. The error bars show the measurement uncertainties.

Droplet	Time	R_p (μm)	R_d (μm)	R (μm)	δ_1 (μm)	δ_2 (μm)	δ (μm)	a (μm)	a_H (μm)
	Before contact	10.0 ± 0.5	13 ± 1	5.7 ± 0.3	0	0	0	0	0
1	Right after contact	10.0 ± 0.5	13 ± 1	5.7 ± 0.3	3.5 ± 0.5	0.2 ± 0.5	3.7 ± 0.7	4.7 ± 1.0	4.6 ± 0.9
	7s after contact	10.0 ± 0.5	13 ± 1	5.7 ± 0.3	3.5 ± 0.5	0.3 ± 0.5	3.8 ± 0.7	4.8 ± 1.0	4.7 ± 0.9
	Before contact	10.0 ± 0.5	14 ± 1	5.7 ± 0.3	0	0	0	0	0
2	Right after contact	10.0 ± 0.5	14 ± 1	5.7 ± 0.3	3.0 ± 0.5	0.8 ± 0.5	3.8 ± 0.7	5.5 ± 1.0	4.7 ± 0.9
	7s after contact	10.0 ± 0.5	14 ± 1	5.7 ± 0.3	3.0 ± 0.5	1.0 ± 0.5	4.0 ± 0.7	5.3 ± 1.0	4.8 ± 0.9
	Before contact	10.0 ± 0.5	13 ± 1	5.6 ± 0.3	0	0	0	0	0
3	Right after contact	10.0 ± 0.5	13 ± 1	5.6 ± 0.3	3.5 ± 0.5	0.5 ± 0.5	4.0 ± 0.7	5.3 ± 1.0	4.7 ± 0.9
	7s after contact	10.0 ± 0.5	13 ± 1	5.6 ± 0.3	3.5 ± 0.5	0.8 ± 0.5	4.3 ± 0.7	5.0 ± 1.0	4.9 ± 0.9

TABLE S2. **Time dependence of the geometry parameters at the probe-droplet contact.** Comparison of the eight geometry parameters: R_p, R_d, R, δ_1, δ_2, δ, a and a_H, obtained at three different times: before the contact, right after the contact, and 7 seconds after the contact. The error bars show the measurement uncertainties.

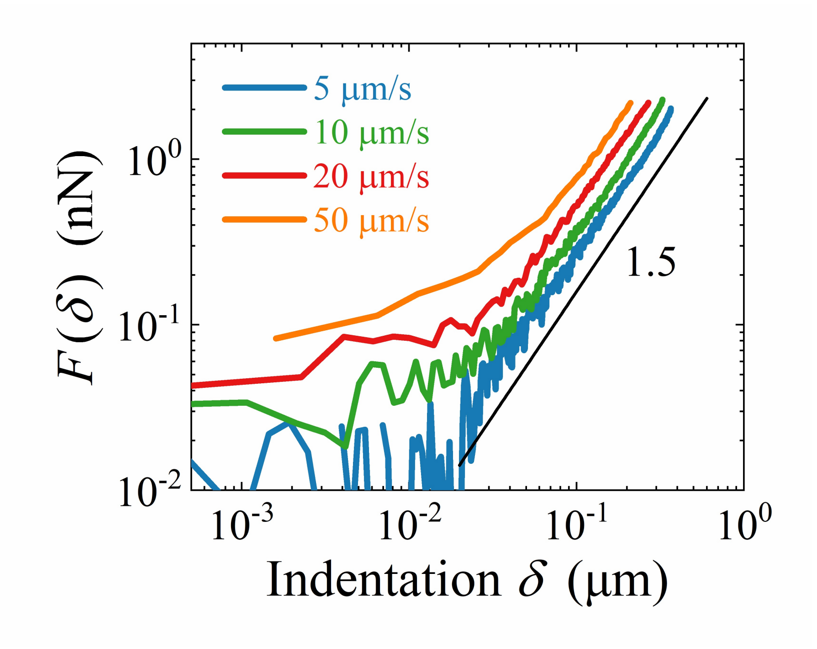

FIG. S12. **Log-log plot of the force-indentation curves.** Replot of the measured force-indentation curves $F(\delta)$ at different loading speeds in the log-log scale. The data are taken from Fig. 1**h** in the main text. The black solid line indicates the Hertz scaling law $F \propto \delta^{3/2}$.

D. Additional measurements for Sample S1

1. *Effects of surface adhesion*

In the discussion of our mechanical model as outlined in Eqs. (1)–(3) of the main text, we only considered the bulk property of the 6xPSD droplets and did not include the effect of surface adhesion between the probe and droplet surface. Herein, we discuss the possible effects of surface adhesion on the measured force relaxation and modulus amplitude E_0. It is seen from Fig. 1**h** in the main text that the force acting on a 6xPSD droplet becomes negative as the probe retracts from the droplet surface, indicating a measurable surface adhesion. The minimal value of the retraction force is called the pull-off force $F_{\rm adh}$ (see Fig. S14(A) below) [30–32]. Although the colloidal probe is coated with a thin layer of PLL-g-PEG to reduce the surface adhesion (see Methods section in the main text for more details), the obtained value of $F_{\rm adh}$ for the 6xPSD droplet still remains a fraction of the maximal applied force F_0. The actual value of $F_{\rm adh}$ is found to depend on a number of factors, such as the surface treatment of the probe, the applied maximal force F_0, and the hold time $t_{\rm dwell}$ used in the stage (ii) of the relaxation measurement before retraction (see Fig. 1**d** in the main text). In the following, we show that the obtained relaxation modulus $E(t)$ and its initial value E_0 are rarely affected by the surface adhesion.

To examine the effect of surface adhesion on the stress relaxation measurements, we systematically varied the surface coating of the AFM probe by a gradual removal of the coating. First, a probe with a fresh PLL-g-PEG coating (PEG-coated) is used to perform the force relaxation measurement (red curve in Fig. S13(A)). Then, the same probe is cleaned with ethanol (EtOH-cleaned) twice to partially remove the PEG-coating and is used to repeat the measurement (blue curve in Fig. S13(A)). Finally, the probe is plasma cleaned for 10 s to remove most of the PEG-coating and is used to repeat the measurement (green curve in Fig. S13(A)). As a result, the adhesion between the probe and droplet surface increases significantly with the reduction of the PEG-coating on the probe, as $F_{\rm adh}$ increases from 3.9 nN to 24.3 nN. It is seen from Fig. S13(A) that the force relaxation curves $F(t)/F_0$ in the three scenarios remain essentially unchanged, indicating that the surface adhesion does not influence the measured $F(t)/F_0$ very much.

Apart from using different probe surfaces, we also investigate whether the measured $F(t)/F_0$ is affected by changing the setpoint force F_0 and the hold time $t_{\rm dwell}$. In the measurements shown in Fig. S13(B), the value of $F_{\rm adh}$ is increased from 19 nN to 53 nN as the maximal applied force F_0 is changed from 2 nN to 8 nN at a fixed $t_{\rm dwell} = 10$ s, yet all the measured force relaxation curves $F(t)/F_0$ overlap with each other and do not change with $F_{\rm adh}$. In the measurements shown in Fig. S13(C), the value of $F_{\rm adh}$ is increased from 5.8 nN to 43 nN as the value of $t_{\rm dwell}$ is changed from 0.1 s to 30 s at a fixed $F_0 = 3$ nN, all the measured force relaxation curves $F(t)/F_0$ remain unchanged. The results shown in Fig. S13 thus demonstrate that the measured force relaxation curves $F(t)/F_0$ are insensitive to the variations of surface adhesion introduced by using different surface treatments and different values of the maximal applied force F_0 and hold time $t_{\rm dwell}$.

To examine the effect of surface adhesion on the force-indentation measurement and determination of the modulus amplitude E_0, we varied the surface coating of the AFM probe, as discussed above. As shown in the left panel of Fig. S14(A), the three surface treatments produce a large change in the shape of the retraction curves and give rise to a large increase of the adhesion force $F_{\rm adh}$. The three approach curves of $F(\delta)$ all show the Hertizan-like behavior, even though they have different apparent moduli. This is caused primarily by sample variations among different batches and by differences in droplet heights (and hence different values of χ, see discussions in Section II A 2). As shown in the right panel of Fig. S14(A), in the normalized plot of $F(\delta)/F_0$ versus δ/δ_0, where δ_0 is the maximum indentation when $F = F_0$, all the approach curves collapse onto a single master curve, whereas the retraction curves show significant dependence on surface adhesion. In the measurements shown in Fig. S14(B), the same PEG-coated probe is used but the maximal applied force F_0 is varied in the range of 1–5 nN, while the other experimental parameters are kept unchanged. Again, in this case, all the normalized approach curves $F(\delta)/F_0$ as a function of δ/δ_0 collapse onto a single master curve, whereas the normalized retraction curves show some differences in $F_{\rm adh}/F_0$. The results shown in Fig. S14 thus demonstrate that the measured force-indentation curves $F(\delta)/F_0$ obtained in the approaching direction are much less sensitive to the variations of surface adhesion compared with those measured in the retraction direction.

Our AFM measurements as described above indicate that the adhesion does not affect the force-indentation curves $F(\delta)$ very much during the loading (advancing) period and only plays a role in the retraction process. As shown in Fig. S12, the measured force-indentation curves $F(\delta)$ during the loading period can all be well described by the Hertz scaling, $F(\delta) \propto \delta^{3/2}$, for different loading speeds. This asymmetric effect was also observed for polyacrylamide hydrogels [33, 34]. While we have not yet found a microscopic theory to explain this asymmetric effect, the experimental observations are nevertheless robust. In fact, if the adhesive force were significant during the loading period, the resulting force-indentation curve $F(\delta)$ would be very different from what we have observed in Fig. S11. An example can be found in Ref [35].

Because the probe-droplet contact remains unchanged with time, as described in Section II C, the measured stress

relaxation $E(t)$ will not be affected by the surface adhesion. This conclusion is further confirmed by the additional measurements described in this subsection. Specifically, we showed in Fig. S13 that the measured force relaxation $F(t)/F_0$ does not change with different surface treatments, the amplitude of the applied force F_0, and the probe holding time t_{dwell}. As shown in Fig. S14, these three parameters have introduced significant changes in adhesion during the retraction process. Nonetheless, the measured force-indentation curves $F(\delta)$ during the loading period do not change with them. Our experiments thus demonstrate that the measured force relaxation $F(t)/F_0$ and force-indentation $F(\delta)$ during the loading period are not influenced by the surface adhesion at the probe-droplet contact, which only plays a role in the retraction process.

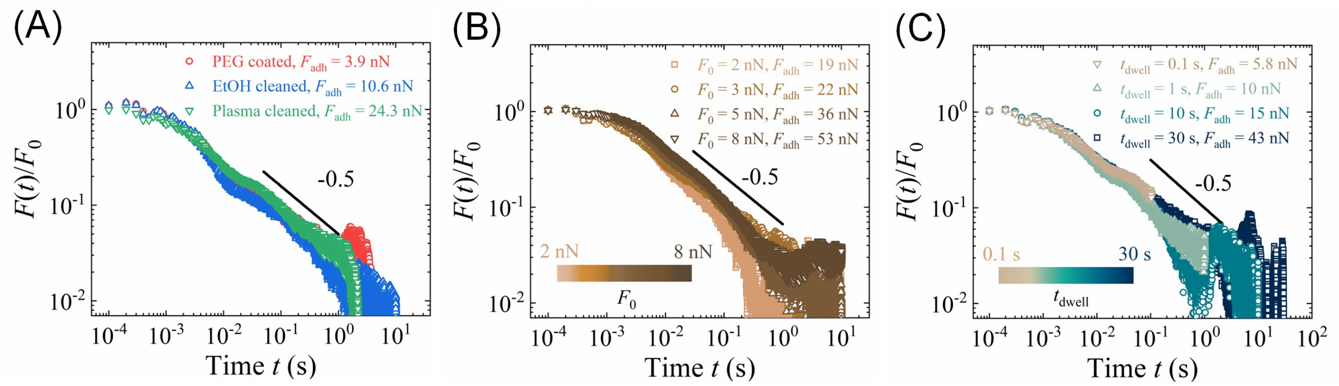

FIG. S13. **Measured force relaxation curves $F(t)/F_0$ with different surface adhesions.** (A) Measured force relaxation curves $F(t)/F_0$ by using a colloidal probe with three different surface treatments: PEG-coated (red circles), EtOH-cleaned (blue upward-triangles) and plasma-cleaned (green downward-triangles). (B) Measured force relaxation curves $F(t)/F_0$ with different setpoint forces F_0 in the range of 2-8 nN. Darker colored curves are obtained by using a larger value of F_0, which gives rise to a larger adhesion force F_{adh}. (C) Measured force relaxation curves $F(t)/F_0$ with different hold times t_{dwell} in the range of 0.1-30 s. Darker colored curves are obtained by using a larger value of t_{dwell}, which gives rise to a larger adhesion force F_{adh}. All of the force relaxation curves are shown in the log-log scale. The black solid lines indicate the power-law decay, $t^{-0.5}$.

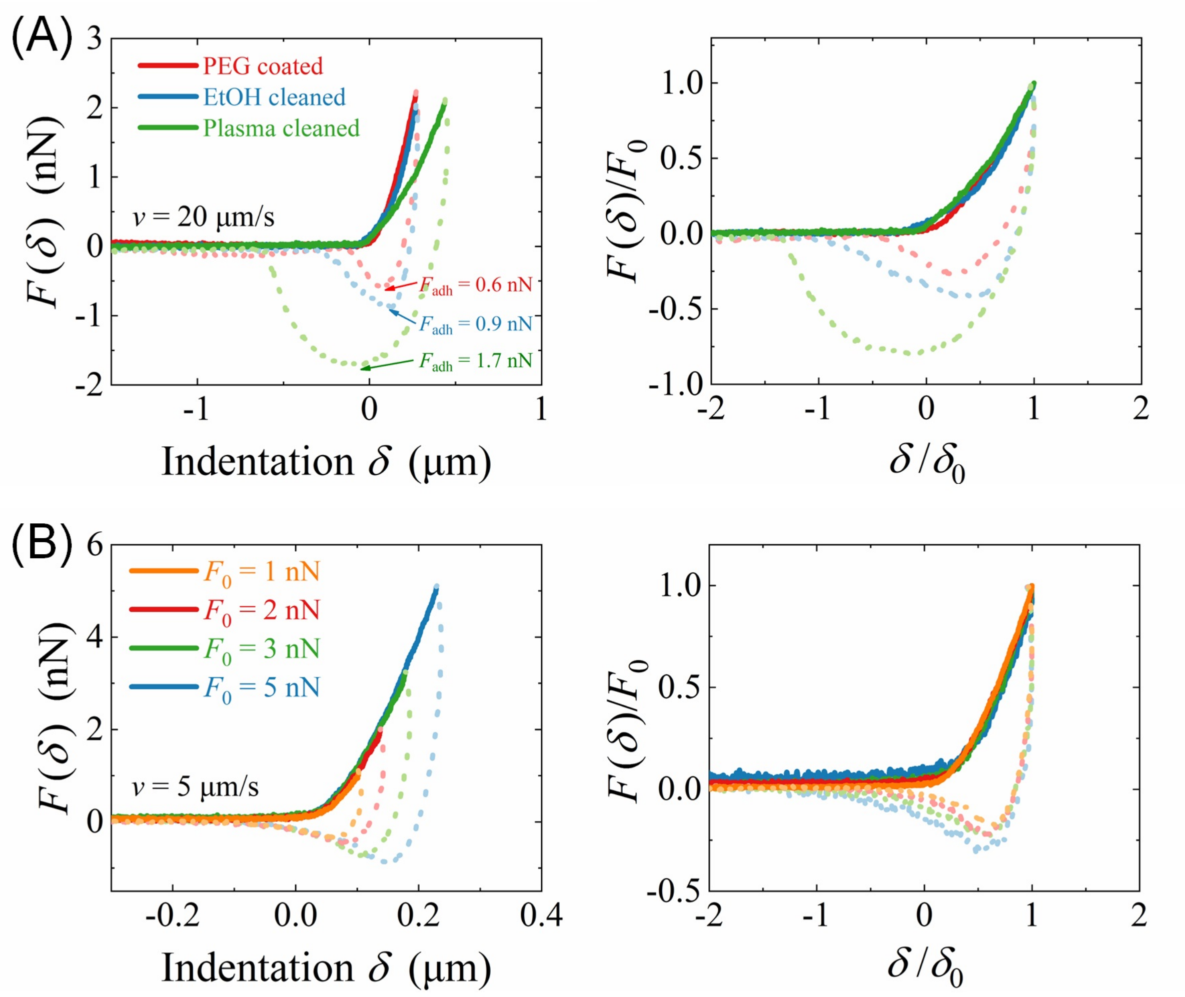

FIG. S14. **Measured force-indentation curves $F(\delta)$ with different surface adhesions.** (A) Measured force-indentation curves $F(\delta)$ by using a colloidal probe with three different surface treatments: PEG-coated (red line), EtOH-cleaned (blue line) and plasma-cleaned (green line). All the measurements are made at the same loading speed $v = 20$ μm/s. The arrows in the left panel point to the adhesion force $F_{\rm adh}$ obtained from the three curves. The right panel shows the normalized plots $F(\delta)/F_0$ as a function of δ/δ_0 with δ_0 being the maximal indentation when F reaches the setpoint force F_0. The data used in the right panel are the same as those in the left panel. (B) Measured force-indentation curves $F(\delta)$ with different setpoint forces: $F_0 = 1$ nN (orange line), 2 nN (red line), 3 nN (green line) and 5 nN (blue line). All the measurements are made at the same loading speed $v = 5$ μm/s. The right panel shows the normalized plots $F(\delta)/F_0$ as a function of δ/δ_0 using the same data as those in the left panel.

2. Hysteresis

From the measured force-indentation loops shown in Fig. 1**h** of the main text, we calculate the hysteresis H_A defined as the area enclosed by the approach and retraction curves in the force-indentation loop (the grey area in Fig. S15(A)). It is seen from Fig. S15(B) that the obtained hysteresis H_A depends very weakly on the indentation speed v, as the mean value of H_A changes only slightly for different values of v. To further verify that the obtained hysteresis H_A is not affected by the variations of the maximal force F_0 applied and the maximal indentation δ_0 achieved during the measurements (see Fig. 1**h** in the main text), we plot, in Fig. S15(C), the normalized hysteresis $H_A/(F_0\delta_0)$ as a function of indentation speed v. It is seen that the mean value of the obtained $H_A/(F_0\delta_0)$ only increases slightly with v.

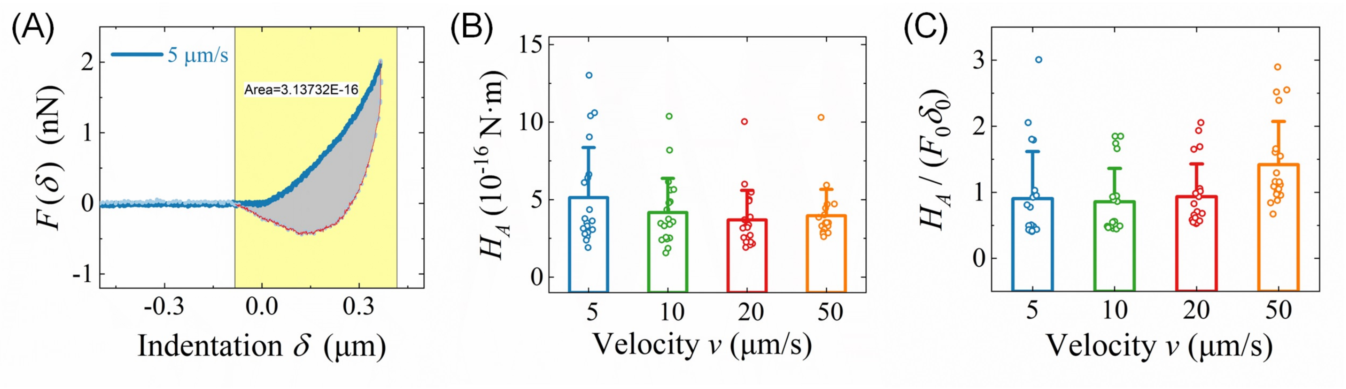

FIG. S15. **Measured hysteresis in the force-indentation loop.** (A) Hysteresis H_A defined as the area enclosed by the approach and retraction curves (grey area) in the force-indentation loop. (B) Measured H_A as a function of indentation speed v. (C) Normalized hysteresis $H_A/(F_0\delta_0)$ as a function of indentation speed v. The individual data points shown in (B) and (C) are obtained from different 6xPSD droplets. The solid symbols show the mean value and the standard deviation of the measurements ($N = 20$ droplets).

3. Effects of modifications to the scaffold protein GKAP

To examine how the individual protein interactions, particularly the weak bonds in the network affect the mechanical behavior of 6xPSD condensate, we made several attempts to alter the binding affinity (or the dissociation constant K_d) between the two scaffold proteins, PSD-95 and GKAP. The binding between PSD-95 and GKAP is driven primarily by the specific interactions between the guanylate kinase (GK) domain in PSD-95 and the GK-binding repeats (GBR) domain in GKAP [36, 37]. With the modifications that target the GBR domain in GKAP, such as phosphorylation and amino acid sequence substitution, one may regulate the interaction strength of the PSD-95/GKAP binding and observe its effects on the condensate mechanics.

First, we tried to phosphorylate the scaffold protein GKAP. Upon phosphorylation of a serine residue in the GBR domain, the binding between the phosphorylated GKAP (pi-GKAP) and PSD-95 is enhanced by about 900-fold compared to the unphosphorylated one, with the PSD-95/GKAP dissociation constant K_d decreased significantly from 176 μM to 0.2 μM [38]. As shown in Fig. S16(A), the measured force relaxation $F(t)/F_0$ (proportional to the time-dependent modulus $E(t)$) for the 6xPSD with pi-GKAP (red circles) changes considerably compared with the control (unphosphorylated, black squares). The relaxation of the 6xPSD with pi-GKAP decays slower than the unphosphorylated one and levels off at large times t. This result suggests that a solid-like permanent network is developed in the condensate so that its modulus $E(t)$ has an asymptotic (constant) value of E_∞ at large t. We speculate that the phosphorylation of GKAP changes the protein-protein interactions so drastically that the network mechanics changes significantly. Thus, the network behavior of the 6xPSD with pi-GKAP is more solid-like than the unphosphorylated one.

Second, we utilized a designed GKAP with its GBR domains substituted by a special amino acid sequence from a peptide DLS that mimics the GKAP phosphorylation (GKAP-DLS). The sequence substitution in GKAP is similar to the phosphorylation of GKAP but affects the PSD-95/GKAP binding in a weaker manner. The binding strength between GKAP-DLS and PSD-95 is increased approximately by 100-fold with its dissociation constant K_d changed from 176 μM to 1.7 μM [38]. As shown in Fig. S16(B), the measured force relaxation $F(t)/F_0$ for the 6xPSD with one DLS substitution (6xPSD-1xDLS, red circles) follows the same two-mode relaxation as the control (black squares). Its power-law relaxation has a slightly smaller exponent α (changed from 0.54 to 0.5) and twice larger relaxation time τ_2 (changed from 2.9 ms to 5.8 ms). When the number of DLS substitutions is increased to three, the binding between GKAP-3xDLS and PSD-95 is further enhanced [38]. The measured $F(t)/F_0$ (6xPSD-3xDLS, blue triangles) decays more slowly and eventually levels off at large times t, similar to the phosphorylation case as shown in Fig. S16(A). When the weakest binding of PSD-95/GKAP is enhanced significantly, other protein interactions may start to play a role in determining the elastic response of the 6xPSD at long times.

The above results provide qualitative support to the proposed mechanism that the power-law response of 6xPSD depends sensitively on the PSD-95/GKAP binding. When the binding strength between GKAP and PSD-95 is enhanced by phosphorylation or sequence substitution, the resulting condensates become more solid-like (e.g., the appearance of a permanent modulus component E_∞ at large times). To test the CGD model more quantitatively, one needs to introduce well-controlled and small perturbations to the PSD-95/GKAP binding at a specific targeted site. The changes induced by phosphorylation and sequence substitution appear so drastic that the intrinsic nature of the protein network is changed from a transient to a more permanent network. As the 6xPSD has multiple protein components and numerous targeted sites for specific protein-protein interactions, finding a precise way to fine-tune the interaction strength at a specific targeted site requires a systematic effort to explore a large parameter space, which demands more work in the future.

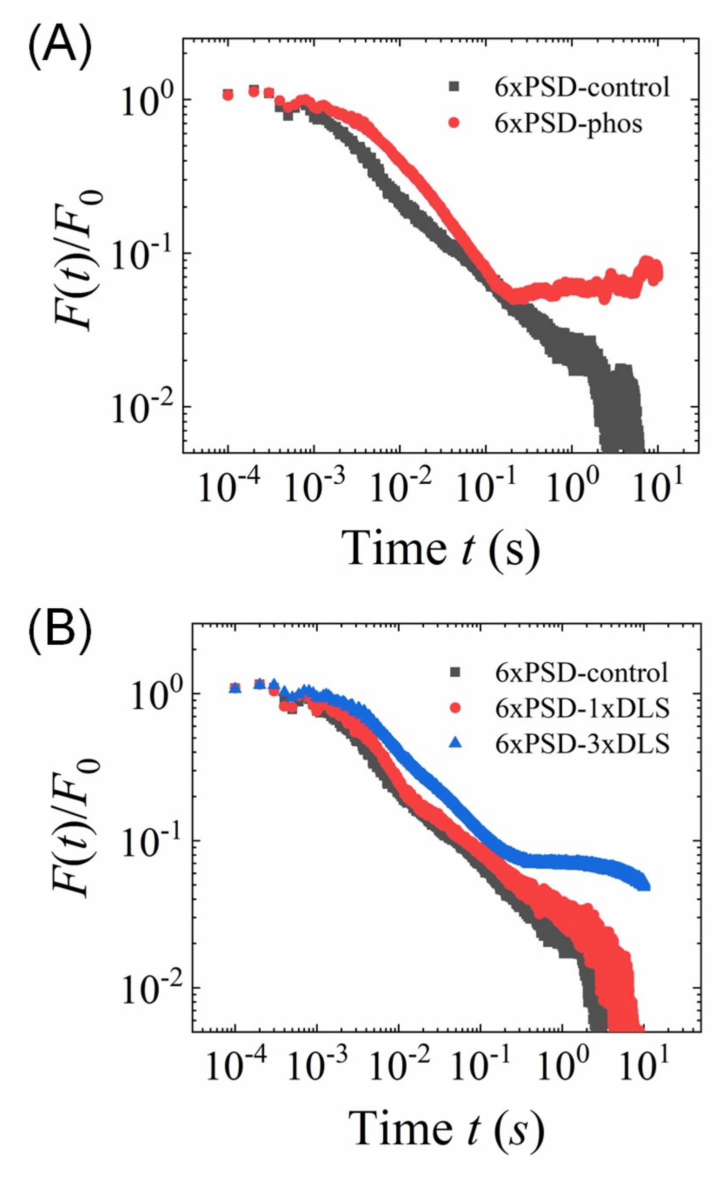

FIG. S16. **Effects of GKAP modifications on the mechanics of 6xPSD condensate.** (A) Comparison of the measured force relaxation curves $F(t)/F_0$ between the 6xPSD with the phosphorylated GKAP (red circles) and the control (unphosphorylated GKAP, black squares). (B) Comparison of the measured $F(t)/F_0$ among the 6xPSD with one DLS substitution in GKAP (6xPSD-1xDLS, red circles), 6xPSD with three DLS substitutions in GKAP (6xPSD-3xDLS, blue triangles), and the control (6xPSD with wild-type GKAP, black squares).

E. Additional measurements for Sample S2

In addition to the mechanical measurements for Sample S1 as reported in Table I of the main text, we also conducted similar measurements for Sample S2. The two samples are very similar, but they were prepared by two different groups of researchers. Sample S2 was prepared for the experiments reported in Ref. [39], and Sample S1 was prepared in this experiment following the same experimental protocols. The only difference between the two samples is that the final concentration of NR2B in S2 is 10 μM instead of 5 μM in S1.

Figure S17(A) shows the measured force relaxation curve $F(t)/F_0$ at the loading speed $v = 100$ μm/s and the data is averaged over 2 droplets of 6xPSD. The measured $F(t)/F_0$ for Sample S2 reveals a short-time exponential decay and a long-time power-law decay until the signal reaches the noise background at $t \sim 2$ s. The crossover time $t_c \simeq 15$ ms (vertical dashed line) indicates that the two relaxation modes are well separated in time. The data is well fitted by Eq. (S7) (blue solid line), further confirming that the two-mode relaxation that well describes the data for Sample S1 also works for Sample S2. The final fitting results are given in Table I of the main text.

Figure S17(B) shows the force-indentation curves $F(\delta)$ obtained from Sample S2 with different loading speeds from $v = 5$ μm/s to 50 μm/s. Similar to Sample S1, the measured $F(\delta)$ for Sample S2 shows a clear speed-dependence during the probe approaching and a strong adhesion during the probe retraction. When the measured $F(\delta)$ during the probe approaching is normalized by the factor $C(t)$ given in Eq. (S8), with the five relaxation parameters: C_1, C_2, τ_1, τ_2, and α, obtained from Fig. S17(A), all of the force-indentation curves collapse onto a master curve, as shown in Fig. S17(C). The obtained master curve is well described by Eq. (S8) (black dashed line) with a single fitting parameter $E_0 = 6904$ Pa. The results shown in Fig. S17 thus demonstrate that the two-mode relaxation modulus $E(t)$, which results from the viscoelasticity of the 6xPSD samples, can be fully determined by the combined measurements of stress relaxation and force indentation relation.

Both sets of data from Samples S1 and S2 confirm that the mechanical properties of the 6xPSD condensates are well described by the two-mode relaxation modulus $E(t)$ as shown in Eq. (S7). There are some slight differences in the fitting parameters, as shown in Table I of the main text, which are caused primarily by the slow aging effect of the protein samples.

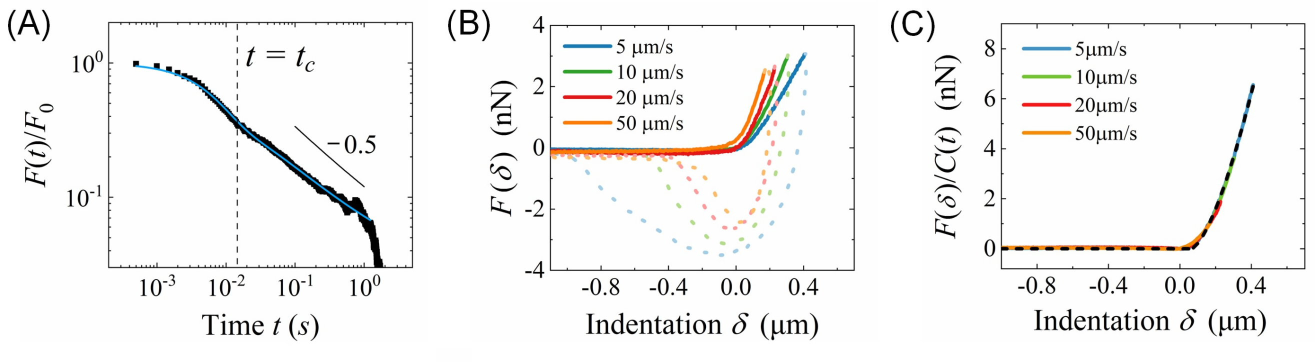

FIG. S17. **Stress relaxation and force-indentation relation obtained from Sample S2.** (A) Log-log plot of the normalized force relaxation $F(t)/F_0$ as a function of time t for Sample S2. The blue solid line shows a fit of Eq. (S7) to the data points. The crossover time $t_c \simeq 15$ ms (vertical dashed line) indicates the temporal separation of the short-time exponential decay from the long-time power-law decay. The black solid line indicates the power-law decay, $t^{-0.5}$. (B) Measured force-indentation curves $F(\delta)$ as a function of indentation δ during the probe approaching (colored solid lines) and probe retraction (colored dotted lines). The measurements are made at different loading speeds: $v = 5$ μm/s (blue), 10 μm/s (green), 20 μm/s (red) and 50 μm/s (orange). (C) Normalized force-indentation curves $F(\delta)/C(t)$ during the probe approaching with different loading speeds v. The data are taken from (B) with the same color codes. The black dashed line is a fit of Eq. (S8) to the data points with a single fitting parameter $E_0 = 6904$ Pa.

III. SUPPLEMENTARY REFERENCES

[1] Taylor, N. O., Wei, M.-T., Stone, H. A., & Brangwynne, C. P. (2019). Quantifying Dynamics in Phase-Separated Condensates Using Fluorescence Recovery after Photobleaching. *Biophys. J.*, 117(7), 1285–1300.
[2] Soumpasis, D. M. (1983). Theoretical analysis of fluorescence photobleaching recovery experiments. *Biophys. J.*, 41(1), 95–97.
[3] Guan, D., Shen, Y., Zhang, R., Huang, P., Lai, P.-Y., & Tong, P. (2021). Unified description of compressive modulus revealing multiscale mechanics of living cells. *Phys. Rev. Research*, 3(4), 043166.
[4] Ting, T. C. T. (1966). The Contact Stresses Between a Rigid Indenter and a Viscoelastic Half-Space. *J. Appl. Mech.*, 33(4), 845–854.
[5] Garcia, P. D., Guerrero, C. R., & Garcia, R. (2020). Nanorheology of living cells measured by AFM-based force–distance curves. *Nanoscale*, 12(16), 9133–9143.
[6] Hertz, H. (1881). On the contact of elastic solids. *J. Reine Angew. Math.*, 92, 156-171.
[7] Dimitriadis, E. K., Horkay, F., Maresca, J., Kachar, B., & Chadwick, R. S. (2002). Determination of Elastic Moduli of Thin Layers of Soft Material Using the Atomic Force Microscope. *Biophys. J.*, 82(5), 2798–2810.
[8] Chadwick, R. S. (2002). Axisymmetric Indentation of a Thin Incompressible Elastic Layer. *SIAM J. Appl. Math.*, 62(5), 1520–1530.
[9] Richbourg, N. R., & Peppas, N. A. (2020). The swollen polymer network hypothesis: Quantitative models of hydrogel swelling, stiffness, and solute transport. *Prog. Polym. Sci.*, 105, 101243.
[10] Tsuji, Y., Li, X., & Shibayama, M. (2018). Evaluation of Mesh Size in Model Polymer Networks Consisting of Tetra-Arm and Linear Poly(ethylene glycol)s. *Gels*, 4(2), 50.
[11] Wisniewska, M. A., Seland, J. G., & Wang, W. (2018). Determining the scaling of gel mesh size with changing crosslinker concentration using dynamic swelling, rheometry, and PGSE NMR spectroscopy. *J. Appl. Polym. Sci.*, 135(45), 46695.
[12] Dai, Y., You, L., & Chilkoti, A. (2023). Engineering synthetic biomolecular condensates. *Nat. Rev. Bioeng.*, 1(7), 466–480.
[13] Doi, M. (2013). *Soft Matter Physics.* Oxford University Press.
[14] Jawerth, L., Fischer-Friedrich, E., Saha, S., Wang, J., Franzmann, T., Zhang, X., Sachweh, J., Ruer, M., Ijavi, M., Saha, S., Mahamid, J., Hyman, A. A., & Jülicher, F. (2020). Protein condensates as aging Maxwell fluids. *Science*, 370(6522), 1317–1323.
[15] Wang, Z., Chen, D., Guan, D., Liang, X., Xue, J., Zhao, H., ... & Zhang, H. (2022). Material properties of phase-separated TFEB condensates regulate the autophagy-lysosome pathway. *J. Cell Biol.*, 221(5), e202112024.
[16] University of Glasgow Research Data [https://researchdata.gla.ac.uk/1604/]
[17] Chim, Y. H., Mason, L. M., Rath, N., Olson, M. F., Tassieri, M., & Yin, H. (2018). A one-step procedure to probe the viscoelastic properties of cells by Atomic Force Microscopy. *Sci. Rep.*, 8(1), 14462.
[18] Moreno-Guerra, J. A., Romero-Sánchez, I. C., Martinez-Borquez, A., Tassieri, M., Stiakakis, E., & Laurati, M. (2019). Model-free rheo-AFM probes the viscoelasticity of tunable DNA soft colloids. *Small*, 15(42), 1904136.
[19] Rubinstein, M., & Colby, R. H. (2003). *Polymer physics.* Oxford university press.
[20] Saha, S., Weber, C. A., Nousch, M., Adame-Arana, O., Hoege, C., Hein, M. Y., Osborne-Nishimura, E., Mahamid, J., Jahnel, M., Jawerth, L., Pozniakovski, A., Eckmann, C. R., Jülicher, F., & Hyman, A. A. (2016). Polar Positioning of Phase-Separated Liquid Compartments in Cells Regulated by an mRNA Competition Mechanism. *Cell*, 166(6), 1572-1584.e16.
[21] Hubatsch, L., Jawerth, L. M., Love, C., Bauermann, J., Tang, T. D., Bo, S., Hyman, A. A., & Weber, C. A. (2021). Quantitative theory for the diffusive dynamics of liquid condensates. *eLife*, 10, e68620.
[22] Sollich, P., Lequeux, F., Hébraud, P., & Cates, M. E. (1997). Rheology of Soft Glassy Materials. *Phys. Rev. Lett.*, 78(10).
[23] Broedersz, C. P., Depken, M., Yao, N. Y., Pollak, M. R., Weitz, D. A., & MacKintosh, F. C. (2010). Cross-Link-Governed Dynamics of Biopolymer Networks. *Phys. Rev. Lett.*, 105(23), 238101.
[24] Moeendarbary, E., Valon, L., Fritzsche, M., Harris, A. R., Moulding, D. A., Thrasher, A. J., Stride, E., Mahadevan, L., & Charras, G. T. (2013). The cytoplasm of living cells behaves as a poroelastic material. *Nat. Mater.*, 12(3), 253–261.
[25] Detournay, E., & Cheng, A. H. D. (1993). Fundamentals of poroelasticity. *Analysis and design methods.* pergamon, 113-171.
[26] Li, H., Lian, X., & Guan, D. (2023). Crossover behavior in stress relaxations of poroelastic and viscoelastic dominant hydrogels. *Soft Matter*, 19(29), 5443–5451.
[27] Semenov, A. N., & Rubinstein, M. (1998). Thermoreversible gelation in solutions of associative polymers. 1. Statics. *Macromolecules*, 31(4), 1373-1385.
[28] Stamenović, D. (2006). Two regimes, maybe three? *Nat. Mater.*, 5(8), 597–598.
[29] Broedersz, C. P., & MacKintosh, F. C. (2014). Modeling semiflexible polymer networks. *Rev. Mod. Phys.*, 86(3), 995–1036.
[30] Ebenstein, D. M., & Wahl, K. J. (2006). A comparison of JKR-based methods to analyze quasi-static and dynamic indentation force curves. *J. Colloid Interface Sci.*, 298(2), 652–662.
[31] Johnson, K. L., Kendall, K., & Roberts, A. (1971). Surface energy and the contact of elastic solids. *Proc. R. Soc. Lond. A*, 324(1558), 301-313.
[32] Barber, J. R. (2018). *Contact Mechanics* (Vol. 250) (Springer International Publishing).

[33] Lai, Y., He, D.-J., Hu, Y.-H. (2019). Indentation adhesion of hydrogels over a wide range of length and time scales. *Extreme Mech. Lett.* **31**, 100540.

[34] Lai, Y. & Hu, Y.-H. (2021). The relation between adhesion properties and network properties of hydrogels: A study based on an indentation adhesion method. *Mech. Mater.* **159**, 103877.

[35] Pham, J. T., Schellenberger, F., Kappl, M., & Butt H.-J., (2017). From elasticity to capillarity in soft materials indentation.*Phys. Rev. Mater.* **1**, 015602.

[36] Kim, E., Naisbitt, S., Hsueh, Y.-P., Rao, A., Rothschild, A., Craig, A. M., & Sheng, M. (1997). GKAP, a Novel Synaptic Protein That Interacts with the Guanylate Kinase-like Domain of the PSD-95/SAP90 Family of Channel Clustering Molecules. *J. Cell Biol.*, 136(3), 669–678.

[37] Zhu, J., Zhou, Q., Shang, Y., Li, H., Peng, M., Ke, X., Weng, Z., Zhang, R., Huang, X., Li, S. S. C., Feng, G., Lu, Y., & Zhang, M. (2017). Synaptic Targeting and Function of SAPAPs Mediated by Phosphorylation-Dependent Binding to PSD-95 MAGUKs. *Cell Rep.*, 21(13), 3781–3793.

[38] Wu, H., Chen, X., Shen, Z., Li, H., Liang, S., Lu, Y., & Zhang, M. (2024). Phosphorylation-dependent membraneless organelle fusion and fission illustrated by postsynaptic density assemblies. *Mol. Cell*, 84(2), 309-326.e7.

[39] Zeng, M., Chen, X., Guan, D., Xu, J., Wu, H., Tong, P., & Zhang, M. (2018). Reconstituted Postsynaptic Density as a Molecular Platform for Understanding Synapse Formation and Plasticity. *Cell*, 174(5), 1172-1187.e16.

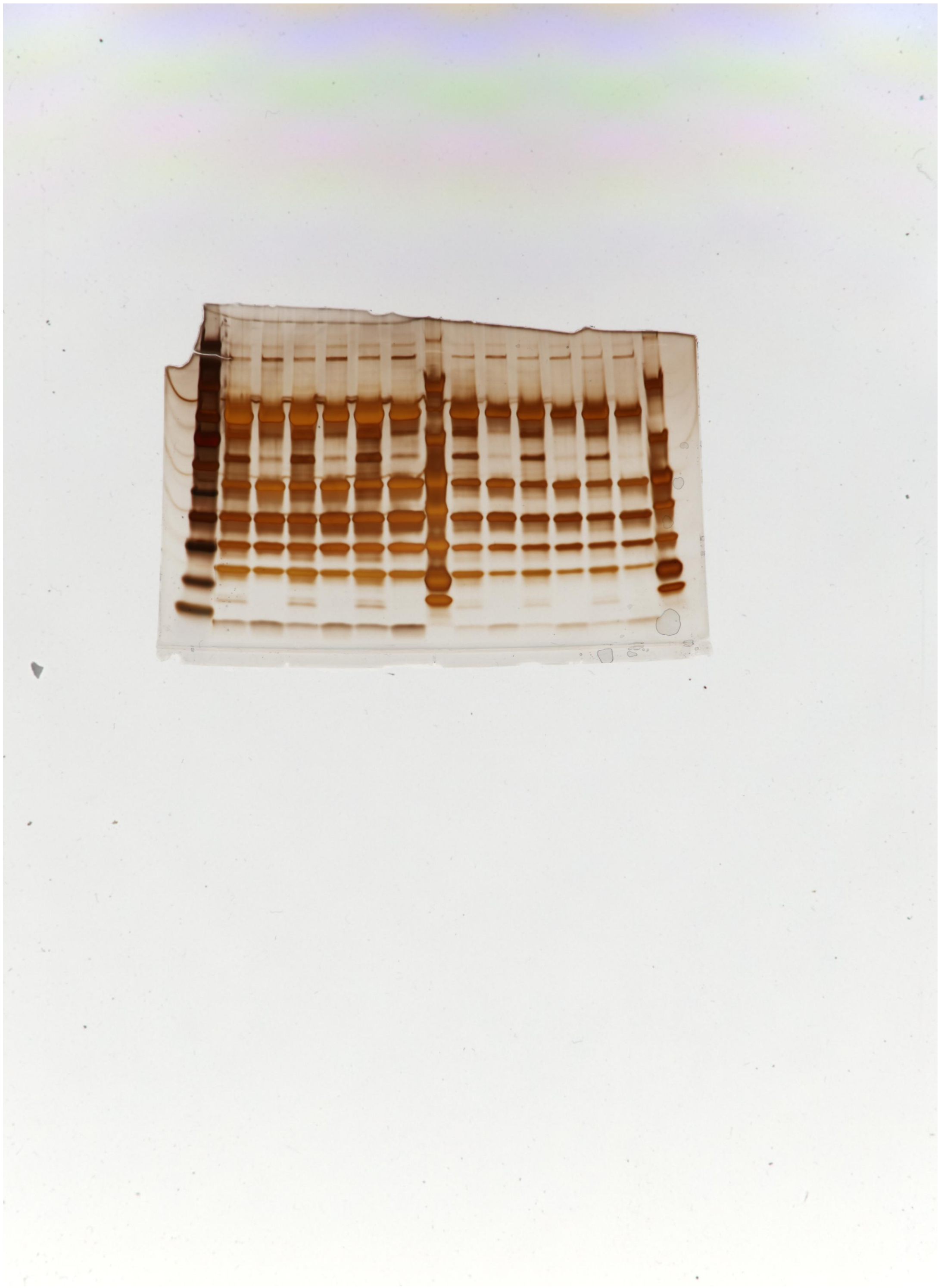

FIG. S18. **Uncropped scan of the gel sample shown in Fig. S5.**
