# Supplementary material for: Emergent mechanics of a networked multivalent protein condensate
Source: Nat Commun. 2025 Jun 5;16:5237. doi: 10.1038/s41467-025-60345-9 (PMC12141447; doi:10.1038/s41467-025-60345-9)
Supplement: Supplementary file 2 — Reporting Summary [file 41467_2025_60345_MOESM2_ESM.pdf]

nature portfolio

Corresponding author(s): Penger Tong, Mingjie Zhang, Dongshi Guan

Last updated by author(s): May 3, 2025

Reporting Summary

Nature Portfolio wishes to improve the reproducibility of the work that we publish. This form provides structure for consistency and transparency in reporting. For further information on Nature Portfolio policies, see our Editorial Policies and the Editorial Policy Checklist.

Statistics

For all statistical analyses, confirm that the following items are present in the figure legend, table legend, main text, or Methods section.

n/a	Confirmed	
☐	☒	The exact sample size (*n*) for each experimental group/condition, given as a discrete number and unit of measurement
☐	☒	A statement on whether measurements were taken from distinct samples or whether the same sample was measured repeatedly
☒	☐	The statistical test(s) used AND whether they are one- or two-sided<br>*Only common tests should be described solely by name; describe more complex techniques in the Methods section.*
☒	☐	A description of all covariates tested
☒	☐	A description of any assumptions or corrections, such as tests of normality and adjustment for multiple comparisons
☐	☒	A full description of the statistical parameters including central tendency (e.g. means) or other basic estimates (e.g. regression coefficient) AND variation (e.g. standard deviation) or associated estimates of uncertainty (e.g. confidence intervals)
☒	☐	For null hypothesis testing, the test statistic (e.g. *F*, *t*, *r*) with confidence intervals, effect sizes, degrees of freedom and *P* value noted<br>*Give P values as exact values whenever suitable.*
☒	☐	For Bayesian analysis, information on the choice of priors and Markov chain Monte Carlo settings
☒	☐	For hierarchical and complex designs, identification of the appropriate level for tests and full reporting of outcomes
☒	☐	Estimates of effect sizes (e.g. Cohen's *d*, Pearson's *r*), indicating how they were calculated

Our web collection on statistics for biologists contains articles on many of the points above.

Software and code

Policy information about availability of computer code

Data collection	MFP3D-IgorPro 14 (AFM software) was used to collect all AFM data. Conventional images were acquired on Nikon Ni-U Upright fluorescent microscope using NIS-Elements BR 5.01.00. Confocal images were acquired on Zeiss LSM880 using Zen2.3 SP1 FP3 V14.0.22.201.
Data analysis	MFP3D-IgorPro 14 was firstly used to process, organize and convert the original AFM data to csv format. The AFM data, FRAP and FIQ images were analyzed (such as normalization, averaging, calculations) by the Matlab 2019a with customed codes. OriginPro 2018 was also used for fitting the data provided with equations. Fiji-ImageJ was used to analyze all graphs. Microsoft Excel was used for data collection and analysis of FIQ and centrifuge assay.

For manuscripts utilizing custom algorithms or software that are central to the research but not yet described in published literature, software must be made available to editors and reviewers. We strongly encourage code deposition in a community repository (e.g. GitHub). See the Nature Portfolio guidelines for submitting code & software for further information.

Data

Policy information about availability of data

All manuscripts must include a data availability statement. This statement should provide the following information, where applicable:

- Accession codes, unique identifiers, or web links for publicly available datasets
- A description of any restrictions on data availability
- For clinical datasets or third party data, please ensure that the statement adheres to our policy

Source data that support the findings of this study (such as figure source data) are provided with this paper in the Source Data file. Unless otherwise stated, all data

supporting the results of this study can be found in the article, supplementary, and source data files. The raw data are available from the corresponding authors upon request. Source Data are provided with this paper.

Research involving human participants, their data, or biological material

Policy information about studies with human participants or human data. See also policy information about sex, gender (identity/presentation), and sexual orientation and race, ethnicity and racism.

Reporting on sex and gender	N/A
Reporting on race, ethnicity, or other socially relevant groupings	N/A
Population characteristics	N/A
Recruitment	N/A
Ethics oversight	N/A

Note that full information on the approval of the study protocol must also be provided in the manuscript.

Field-specific reporting

Please select the one below that is the best fit for your research. If you are not sure, read the appropriate sections before making your selection.

☒ Life sciences ☐ Behavioural & social sciences ☐ Ecological, evolutionary & environmental sciences

For a reference copy of the document with all sections, see nature.com/documents/nr-reporting-summary-flat.pdf

Life sciences study design

All studies must disclose on these points even when the disclosure is negative.

Sample size	No statistical method was used to determine the correct sample size. The sample size is determined based on our experience and our previous studies using similar methodologies (Zeng, M., et al. (2018). Cell, 174(5), 1172-1187; Guan, D., et al. (2021). Phys. Rev. Res., 3(4), 043166.) , which is enough to obtain reliable and consistent results. The exact sample sizes are available from the paper.
Data exclusions	AFM measurements need to be conducted in a noise-free (no vibration) environment and on samples at equilibrium. Some force measurement curves affected by environmental noise and non-steady variations were discarded. This is a standard practice for AFM measurements.
Replication	The main experimental results are all reproducible using different samples and measured on different days. The number of liquid droplets used was indicated in the figure legends and was three or greater for all experiments.
Randomization	No randomization is needed for our experiments.
Blinding	No blinding is needed for this research.

Reporting for specific materials, systems and methods

We require information from authors about some types of materials, experimental systems and methods used in many studies. Here, indicate whether each material, system or method listed is relevant to your study. If you are not sure if a list item applies to your research, read the appropriate section before selecting a response.

Materials & experimental systems

n/a	Involved in the study	
☒	☐	Antibodies
☒	☐	Eukaryotic cell lines
☒	☐	Palaeontology and archaeology
☒	☐	Animals and other organisms
☒	☐	Clinical data
☒	☐	Dual use research of concern
☒	☐	Plants

Methods

n/a	Involved in the study	
☒	☐	ChIP-seq
☒	☐	Flow cytometry
☒	☐	MRI-based neuroimaging

Plants

Seed stocks	N/A
Novel plant genotypes	N/A
Authentication	N/A
